# CellDynaMo–stochastic reaction-diffusion-dynamics model: Application to search-and-capture process of mitotic spindle assembly

**DOI:** 10.1371/journal.pcbi.1010165

**Published:** 2022-06-03

**Authors:** Evgenii Kliuchnikov, Artem Zhmurov, Kenneth A. Marx, Alex Mogilner, Valeri Barsegov

**Affiliations:** 1 Department of Chemistry, University of Massachusetts, Lowell, Massachusetts, United States of America; 2 KTH Royal Institute of Technology, Stockholm, Sweden; 3 Courant Institute for Mathematical Sciences and Department of Biology, New York University, New York, New York, United States of America; National Institutes of Health, UNITED STATES

## Abstract

We introduce a Stochastic Reaction-Diffusion-Dynamics Model (SRDDM) for simulations of cellular mechanochemical processes with high spatial and temporal resolution. The SRDDM is mapped into the CellDynaMo package, which couples the spatially inhomogeneous reaction-diffusion master equation to account for biochemical reactions and molecular transport within the Langevin Dynamics (LD) framework to describe dynamic mechanical processes. This computational infrastructure allows the simulation of hours of molecular machine dynamics in reasonable wall-clock time. We apply SRDDM to test performance of the Search-and-Capture of mitotic spindle assembly by simulating, in three spatial dimensions, dynamic instability of elastic microtubules anchored in two centrosomes, movement and deformations of geometrically realistic centromeres with flexible kinetochores and chromosome arms. Furthermore, the SRDDM describes the mechanics and kinetics of Ndc80 linkers mediating transient attachments of microtubules to the chromosomal kinetochores. The rates of these attachments and detachments depend upon phosphorylation states of the Ndc80 linkers, which are regulated in the model by explicitly accounting for the reactions of Aurora A and B kinase enzymes undergoing restricted diffusion. We find that there is an optimal rate of microtubule-kinetochore detachments which maximizes the accuracy of the chromosome connections, that adding chromosome arms to kinetochores improve the accuracy by slowing down chromosome movements, that Aurora A and kinetochore deformations have a small positive effect on the attachment accuracy, and that thermal fluctuations of the microtubules increase the rates of kinetochore capture and also improve the accuracy of spindle assembly.

## Author summary

The CellDynaMo package models, in 3D, any cellular subsystem where sufficient detail of the macromolecular players and the kinetics of relevant reactions are available. The package is based on the Stochastic Reaction-Diffusion-Dynamics model that combines the stochastic description of chemical kinetics, Brownian diffusion-based description of molecular transport, and Langevin dynamics-based representation of mechanical processes most pertinent to the system. We apply the model to test the Search-and-Capture mechanism of mitotic spindle assembly. We find that there is an optimal rate of microtubule-kinetochore detachments which maximizes the accuracy of chromosome connections, that chromosome arms improve the attachment accuracy by slowing down chromosome movements, that Aurora A kinase and kinetochore deformations have small positive effects on the accuracy, and that thermal fluctuations of the microtubules increase the rates of kinetochore capture and also improve the accuracy.

## Introduction

**Fig 1:**
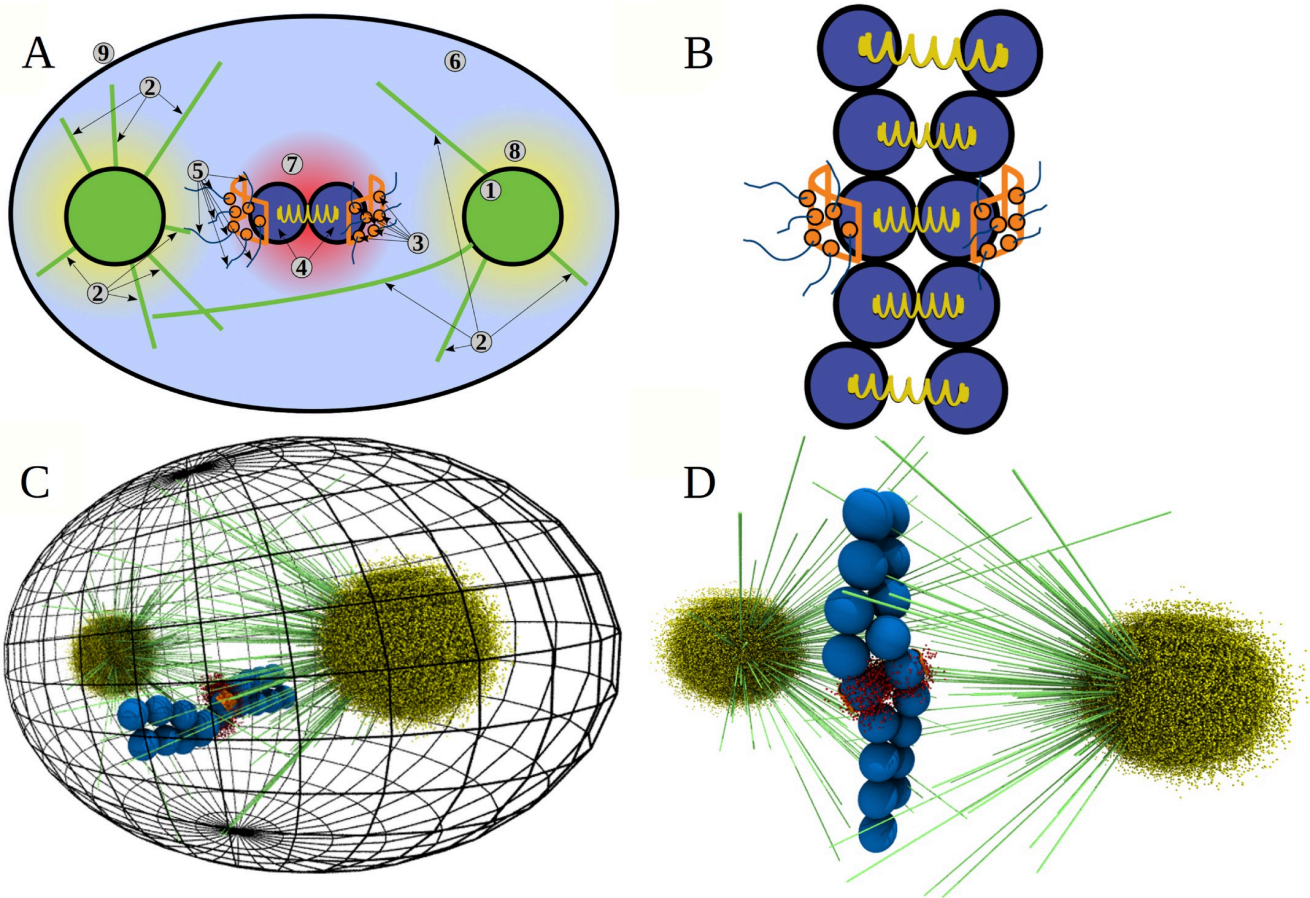
Components of Stochastic Reaction-Diffusion-Dynamics model implemented in CellDynaMo package. **A)** Schematic of the Stochastic Reaction-Diffusion-Dynamics model. 1) Spherical centrosomes (CS) with the microtubules’ (MTs) plus-ends on the surface (400-nm radius, 750 MTs per centrosome); 2) Microtubules (MTs) described by 12.0-nm spherical beads, each representing ~40 4.0-nm αβ-tubulin dimers; 3) Kinetochores’ corona regions (on spherical surface) in which the complexes of Ndc80 bound to MTs with their kinetochore-associated domains, spherical 8.0-nm beads, are labelled; 4) Kinetochore pairs (KTs) and sister Chromatids (Chs) represented by spherical beads with 362.5-nm radius (number of beads depends on the size of the chromatid arms, see **B**); 5) Ndc80 protein complexes with KT via the spherical kinetochore binding domains and the labelled 60.0-nm triple-helical coiled coil domain modeled as a harmonic spring connected to corona surface from one side and to MT plus-end from other side; 6) Blue space is Phosphatase (enzyme performing dephosphorylation of Ndc80), which is uniformly distributed in the interior space of the cell; 7) Aurora B kinase (AB; enzyme performing phosphorylation of Ndc80) is described by the spherical gradient of its concentration with the central maximum in the space between the kinetochores (red cloud); 8) Aurora A kinase (AA; enzyme performing phosphorylation of Ndc80) is described by the spherical gradient of its concentration with the twin maxima centered on the two centrosomes (yellow cloud). In the model, components 1–4 are described using the Langevin Dynamics in the Brownian diffusion limit (Table 1), whereas components 5–8 are modeled using the stochastic reaction-diffusion master equation (Table 2). 9) Cellular membrane is modeled parametrically as a smooth repulsive potential for all cell components. **B)** More detailed view of metaphase chromosomes (CHs) comprised of identical paired sister Chromatids (Chs). The entire length of each chromatid is represented by connected beads and neighboring beads on the adjacent sister chromatids are connected by a spring. **C)** Snapshot in 3D of a single trajectory from the CellDynaMo simulation that shows all the components, including cell membrane (black grid). **D)** Closer cell interior view of the Stochastic Reaction-Diffusion-Dynamics model showing all components: MTs (lime tubes), KTs and Chs (blue beads), corona with Ndc80 seeds (orange wall sections). CS pair are hidden behind point clouds of AA kinase (yellow). AB kinase (red point cloud) is located between KTs.

Cell biological phenomena are governed by far-from-equilibrium dynamically coupled mechanical, chemical, and transport processes that occur in complex cellular morphologies on multiple temporal and spatial scales [1]. A prime example is the assembly of the mitotic spindle in prometaphase, in which chromosomes are mechanically aligned at the spindle equator and sister chromatids are connected to the opposite spindle poles [2] (Fig 1), which enables the spindle to segregate the identical genetic material to opposite parts of the cell during later stages of mitosis.

**Table 1:** Components of CellDynaMo described with Langevin Dynamics approach. Size, shape, and number of each component described using the Langevin Dynamics description of stochastic processes, including centrosomes (CS), microtubules (MTs), kinetochore (KT) corona, chromosomes (CH), and Ndc80 binding domains (Fig 1). Also provided is the information about force-generating properties, number of particles (spherical beads) used to describe each component, and some additional information such as MT persistence length *l*_*p*_, surface curvature of the KT corona *χ*, number of beads per chromatid *n*, and contour length of a chromatid *L*.

Component	Shape	Amount	Radius/Surface area, nm/μm^2^	Force generation	Additional information
centrosome (CS)	sphere	2 per cell	*R*_*CS*_ = 400.0 nm	no	-
Microtubule (MT)	long filament	750 per CS	*R*_*MT*_ = 12.0 nm	yes	*l*_*p*_ = 4 mm
Kinetochore (KT) corona	curved rectangle	2 per CH	*A*_*KT*_ = 0.15 μm^2^	yes	*χ* = 0–1
chromosome (CH)/ chromatid (Ch)	*X*-shaped/ *n*-bead filament	2 per CH	*R*_*CH*_ = 362.5 nm *R*_*Ch*_ = 362.5 nm	yes/ yes	*L* = 4–8 μm *n* = 5–9
Ndc80 binding domain	spherical bead	~750 per KT	*R*_*Ndc*_ = 4.0 nm	yes	-

**Table 2:** Components of CellDynaMo described with Reaction-Diffusion Master Equation formalism. Functional role and the number of copies of each component described with RDME, including MT-Ndc80 complex, Phosphatase enzyme (PH), Aurora A enzyme (AA), Aurora B enzyme (AB), and cellular membrane. Also provided is the information about the force-generating properties and the distribution of each component inside the cell.

Component	Function	Amount	Force generation	Comments
MT-Ndc80 complex	KT-MT attachment	~750 per KT	no	-
Phosphatase (PH)	Ndc80 dephosphorylation	~10 per KT corona	no	uniform distribution
Aurora A (AA)	Ndc80 phosphorylation	~2.0×10^6^	no	gradient of concentration peaked at CS (0–4000 per KT corona)
Aurora B (AB)	Ndc80 phosphorylation	~2.0×10^3^	no	gradient of concentration peaked at KT (~100 KT corona)
Membrane	cell shape	1	no	sphere, ellipse, cube, rectangle

**Fig 2:**
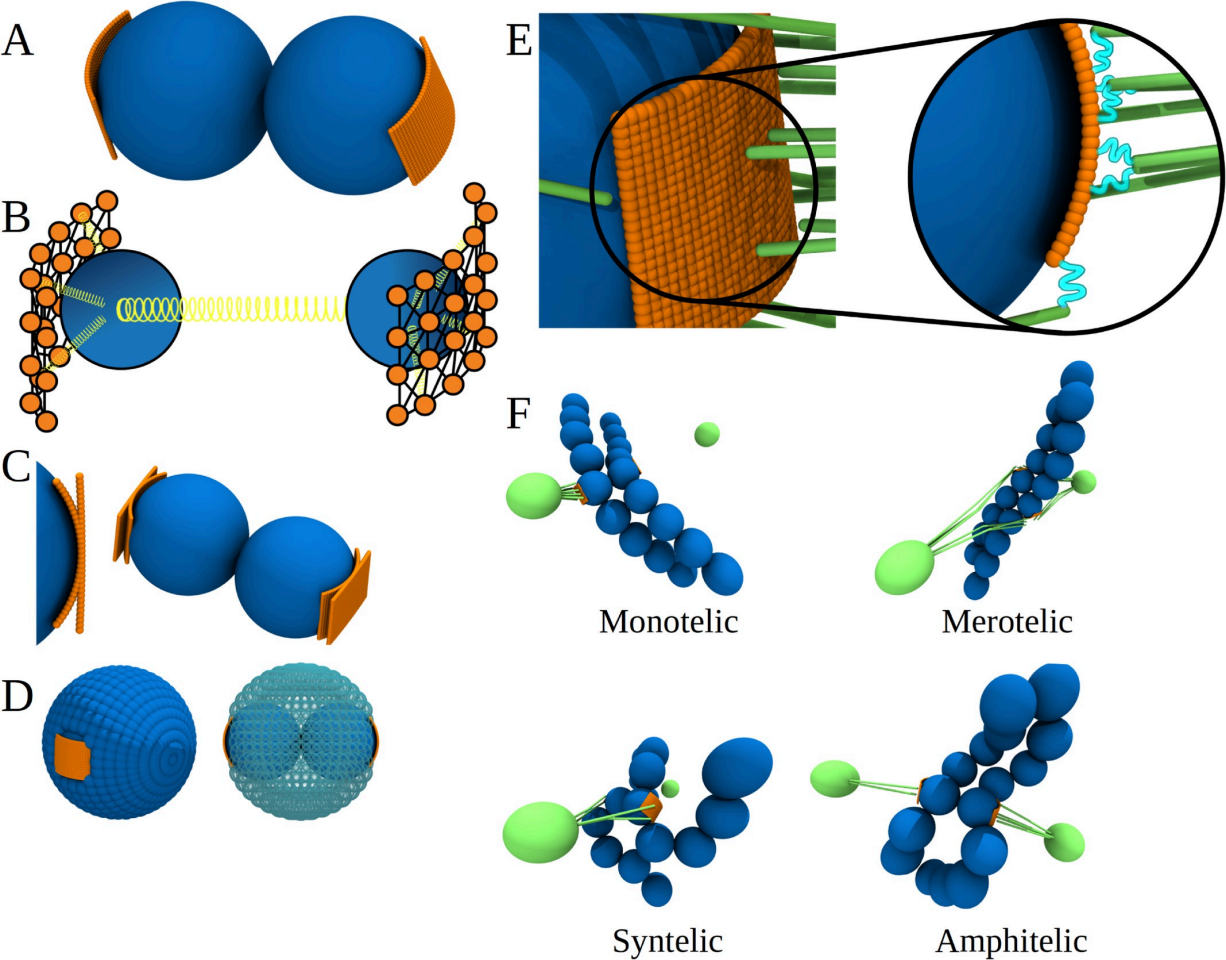
Kinetochore-microtubule attachments in Stochastic Reaction-Diffusion-Dynamics Model. **A**) A regular kinetochore (KT) representation. Blue bead represents the main body of the KT and orange wall section is the outer KT, which is covered by grid of Ndc80 seeds. **B**) Schematic of the KT model. Grid of beads within corona are connected to each other inside some cut-off radius (200 nm); all of them are also attached to the center of mass of the corresponding KT. This set of features was made to keep the KT shape constant. **C**) In the Stochastic Reaction-Diffusion-Dynamics model, we can vary the curvature of the outer KT from the most curved (beads cover the KT surface) to almost flat. **D**) Another option of the model is to cover a KT with molecular “armor” blocking the MT access to KTs, thus mimicking the role of CH arms. **E**) A more detailed look at the KT-MT interface. Here MTs are lime tubes. MTs are attached to the corona with the help of Ndc80-complexes (cyan lines). **F**) Types of KT-MT attachments are illustrated by examples coming from snapshots taken from the simulations.

In animal cells, two centrosomes anchor minus-ends of a microtubule (MT) aster. In prometaphase, dynamic MT plus-ends undergo dynamic instability and become connected to macromolecular structures called kinetochores (KTs) [3] on the opposite ends of the centromere regions of the chromosomes (CHs) (Fig 1). KTs consist of centromere-proximal and centromere-distal protein layers, known as the inner and the outer KT, respectively [4]. The outer KT forms attachments to MT plus-ends through protein linkers called Ndc80 (Figs 1 and 2). In the accurately assembled spindle, one of the sister KTs is connected through multiple MTs to one centrosome (spindle pole), and its sister KT–to the opposite pole; this is the so-called amphitelic, ‘proper’, connection (Fig 2). The affinity of KT-MT attachments depends on the degree of phosphorylation of Ndc80, which is regulated by Aurora kinases, phosphorylating enzymes, and phosphatases, dephosphorylating enzymes. The phosphatase enzymes diffuse in the cytoplasm, while Aurora kinase molecules are tethered to the inner KTs and diffuse to a limited extent in the vicinity of the outer KTs.

The complexity of this multi-component process of the spindle assembly is overwhelming, considering that in addition to the kinetic processes discussed above, pulling forces produced by depolymerizing MTs and molecular motors on the outer KT and pushing by polymerizing MTs on large and deformable CH arms generate chromosome movements [5] and centromere and KT deformations [6]. These movements and deformations feedback to kinetics through multiple molecular pathways. The spindle assembly process unfolds on multiple temporal and spatial scales. For example, transport processes, chemical reactions and MT dynamic instability span over six decades of time from milliseconds (Aurora B diffusion) to centiseconds (MT shortening), to seconds (Ndc80 phosphorylation and dephosphorylation, MT growth), and to minutes (duration of MT catastrophe and rescue). The scale of system lengths spans three orders of magnitude, from a few tens of nanometers (size of Ndc80) to hundreds of nanometers (size of KT), to micrometers (MT length and size of CH), and finally to tens of micrometers (cell size). In addition, low protein copy numbers (e.g. ~10 Phosphatase enzymes per KT corona, hundreds of MTs, ~10^3^ Aurora B enzymes) generate stochastic effects [7]. Therefore, the question is how the seemingly chaotic kinetics and mechanics of tens of CHs and hundreds of MTs result in the rapid formation of very accurate KT-MT connections.

After the discovery of MT dynamic instability, Hill, Kirschner and Mitchison in the mid-eighties proposed an appealing Search-and-Capture hypothesis [8,9], later supported by some experimental observations [10]. According to this hypothesis, MT plus-ends grow and shorten, rapidly, repeatedly and in random directions. When, by chance, the MT plus-end bumps into a KT, a connection is established. In the next decades, the Search-and-Capture model contributed a great deal to our understanding of the mitotic spindle assembly (history and supporting data are reviewed in [11]) by quantitatively examining two key aspects of prometaphase–speed and accuracy. First, the model established that mitosis can be rapid: minutes to tens of minutes are sufficient to connect all CHs to the spindle poles [12]. However, the problem of accuracy remains more elusive. Indeed, since an MT from either pole can attach to any KT in the sister KT pair, a number of possibilities for incorrect KT-MT attachment exist leading to chromosome segregation errors [13] (Fig 2F). In the biologically correct *amphitelic* type of attachment, all MTs connected to each sister KT originate from only one of the two opposite spindle poles (Fig 2). Inaccurate attachments are defined as follows. If only one of two sister KTs is attached to just one spindle pole, this is a *monotelic* attachment. A *syntelic* attachment occurs when both sister KTs are erroneously attached to a single pole and no attachments exist to the second pole. In a *merotelic* attachment, at least one KT is attached to MTs extending from both poles [14] (Fig 2). The earliest simulation studies predicted a majority of merotelic attachments in the absence of any correction mechanisms [15], for the simple reason that, with time, many KTs will be connected to MTs from both poles. Considering the great accuracy of mitosis in healthy cells, the fundamental question is what are the mechanisms that prevent and correct these errors.

Our description of prometaphase is limited–in this study we do not address some key phenomena, such as self-organization of interpolar MT bundles [16], lateral KT-MT connections [17], the action of multiple molecular motors transporting the CHs and organizing the spindle, and alternative, non-centrosomal pathways of spindle assembly [18–20]. Nevertheless, even the over-simplified Search-and-Capture process in animal cells was never simulated within a framework that utilized both realistic geometry and molecular complexity, which is what we aim to accomplish in this study.

The complexity of mitotic spindle assembly described above makes it difficult to explore this Search-and-Capture process using experiments alone, and so many computational models were developed to simulate mitosis [21–26], with a few of them devoted to understanding prometaphase [8,16,27,28]. Two such recent models are the state of the art. In [16,27], Edelmaier et al. simulated the spindle assembly in 3D inside the yeast cell’s nucleus with small MT and CH numbers and simple KT geometry. The important insight was that three error-prevention and correction mechanisms are required for completely correct assembly: 1) stabilization of KT-MT attachment by tension resulting from the KT-KT stretching force, 2) destabilization of misaligned attachments, and 3) progressive angular restriction of attachments. Hypothetical mechanisms 2 and 3 are very restrictive and would require elaborate microscopic molecular processes at KTs. Perhaps the most important lesson from the model in [16,27] is that even in a simplified geometry, achieving accuracy is hard. Another model [28] considered simplified 2D Search-and-Capture of a single CH. Zaytsev et al. demonstrated that, with realistic geometric constraints and rapid MT turnover at KTs, the number of errors can be decreased to a few tens of percent.

**Fig 3:**
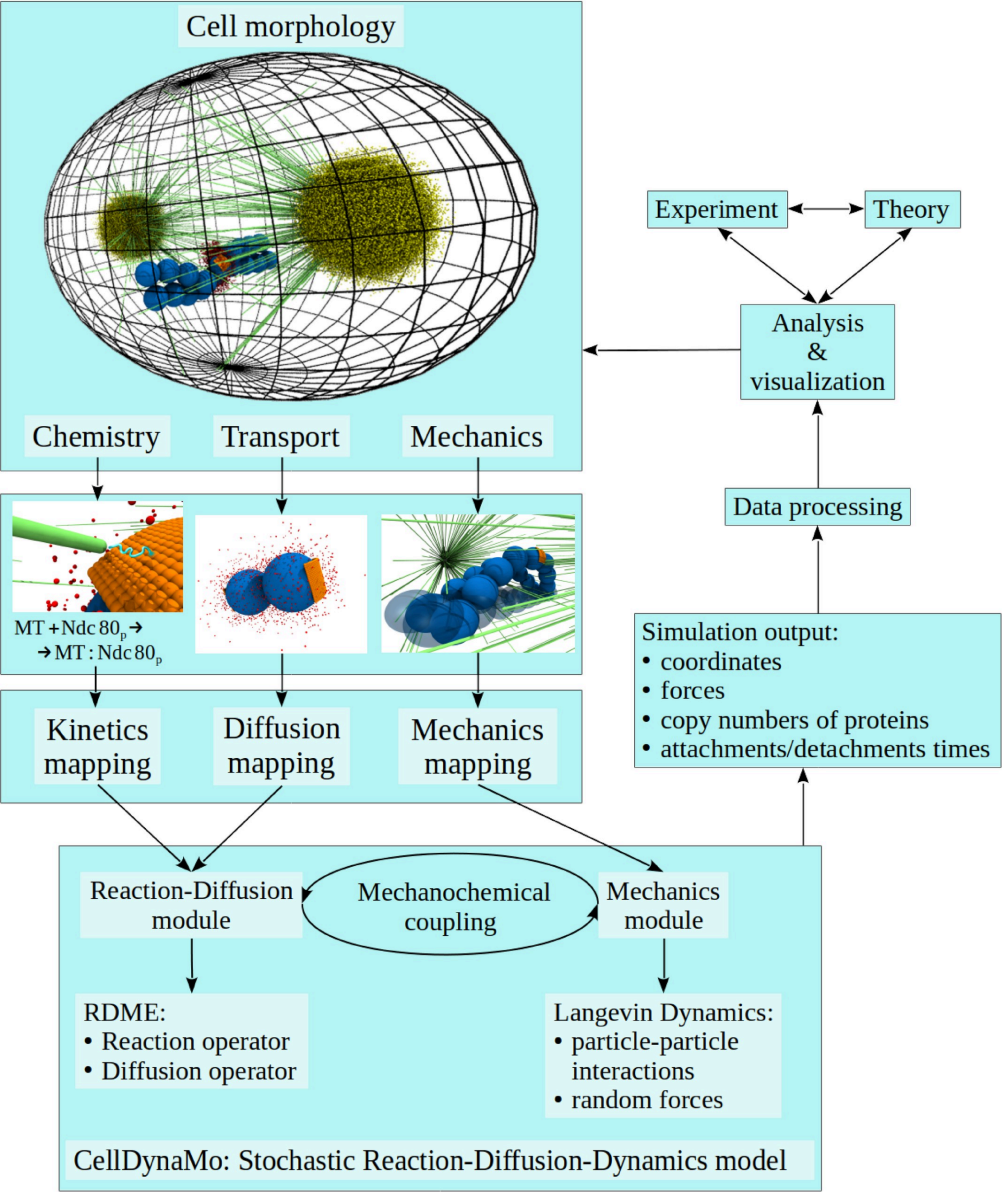
Design of Stochastic Reaction-Diffusion-Dynamics model-based implementation of CellDynaMo package. CellDynaMo requires the initial input (reaction rate constants, copy numbers of biomolecules), the force field parameters (stretching and bending rigidities, KT-MT attachment strength), and cell morphology (number of chromosomes or kinetochore pairs, membrane shape). These specify cell morphology and geometry of spatial arrangements of different cell components (Fig 1), biochemical kinetics (Tables 2, 3 and 4), molecular transport (Table 3), and force-generating properties (Table 1). A list of parameters used in CellDynaMo package is provided in Table A in S1 Text. The RDME is solved numerically for all subcells at each time point. When changes to the mechanical state occur, the RDME switches off and the LD switches on. When a new state of mechanical equilibrium is reached, CellDynaMo writes an output for a particular time point, which includes coordinate file, force file, and file with subcell specific content. These can then be used to analyze and visualize the simulation data, and to compare with experiments and with theoretical predictions.

Our aim here is to simulate the Search-and-Capture process in its full geometric and molecular complexity, going beyond the simplified models described in [15,28], while also not postulating non-molecularly explicit processes as in [16]. In order to follow cell dynamics on spatial scales from tens of nanometers to tens of micrometers and temporal scales from sub-seconds to tens of minutes, we introduce and use the Stochastic Reaction-Diffusion-Dynamics model (SRDDM) that combines the stochastic description of chemical kinetics, Brownian diffusion-based description of molecular transport, and Langevin dynamics-based representation of mechanical processes most pertinent to mitotic spindle assembly (Fig 3). The SRDDM is based on solving the spatially inhomogeneous reaction-diffusion master equation (RDME) for describing the biochemical reactions and molecular transport and propagating the Langevin Dynamics (LD) to treat the dynamic mechanical processes. The cell cytoplasm is divided into discrete subcells (Figs 1 and 2). The molecular species (e.g., Aurora A and Aurora B enzymes) diffuse in and out of these subcells, and react with other molecules (e.g., formation and dissociation of the MT-Ndc80 complex). The RDME formalism [29–33] accounts for biochemical kinetics and molecular transport. The LD approach involves the mechanically coupled elements, including pulling of CHs by shortening MTs, deformation of the CH arms, and restrictions imposed by the cell boundary on MT growth (Fig 1). We also developed a coarse-grained representation and parametrized the force field for the mechanical components involved in mitotic spindle assembly, which include the cell boundary, MTs, KTs, and CH arms. To model the mechanochemistry of mitotic spindle assembly, the RDME formalism and the LD approach are coupled together.

**Table 3:** Microtubule dynamic processes and transport properties of Aurora A and Aurora B enzymes. Dynamic processes involving microtubules (MT) and the values of kinetic rate constants and characteristic timescales associated with the MT growth, shortening, catastrophe and rescue; Δ*l* = 24 nm is the amount by which MT length increases or decreases when MT grows or shortens, respectively. Also shown are the diffusion constants and diffusion timescales for Aurora B and Aurora A enzymes.

Dynamic process	Scheme	Rate/diffusion constant	Timescale
MT growth	${MT}_{l(gr)}\to {MT}_{l+\Delta l(gr)}$	5.0 s^-1^	0.2 s
MT shortening	${MT}_{l(sh)}\to {MT}_{l-\Delta l(sh)}$	18.6 s^-1^	5.4×10^−2^ s
MT catastrophe	${MT}_{l(gr)}\to {MT}_{l(sh)}$	2.5×10^−3^ s^-1^	400.0 s
MT rescue	${MT}_{l(sh)}\to {MT}_{l(gr)}$	3.0×10^−2^ s^-1^	33.3 s
Aurora A/B diffusion	${AB}_{v}({AA}_{v})\to {AB}_{v+\xi }({AA}_{v+\xi })$	7.3×10^7^ nm^2^/s	4.3×10^−4^ s

First, we describe the computational methodology and assess the accuracy of numerical implementation of the RDME formalism and LD approach. Next, we explore the Search-and-Capture process computationally, adding, for the first time, the following important features of animal cells: 1) realistic 3D geometry of KTs and CH arms; 2) instead of idealized attached/detached KT-MT states, we consider molecularly explicit KT-MT connections through elastic Ndc80 linkers; 3) kinetics of KT-MT attachments and detachments mediated by the phosphorylation state of Ndc80 linkers; 4) diffusion and kinetics of Aurora B and phosphatase molecules, which govern the phosphorylation state of Ndc80 linkers; 5) elastic deformation of KTs. This allows us to answer the following questions about the Search-and-Capture process of spindle assembly: What spindle accuracy level can be achieved with the simplified Search-and-Capture process? Are MT dynamics optimal for the accuracy? What are effects of CH arms and thermal fluctuations on the accuracy?

## Methods and models

### Stochastic reaction-diffusion-dynamics model of mitotic spindle assembly

#### Mechanically active components

A schematic of the Stochastic Reaction-Diffusion-Dynamics model is presented in Fig 1. The model includes the following components: centrosomes (spindle poles), microtubules (MT), sister kinetochore (KT) pairs on centromeric region of chromosomes (CHs), chromosome arms, and Ndc80 linkers anchored at the KTs. All these components (see Table 1) are described using Langevin Dynamics in the Brownian diffusion limit. To represent these components, we utilized single interaction centers (beads, spherical particles) or several interaction centers (bead-spring representation) with realistic size and shape. The model has two spherically shaped centrosomes with the radius *R*_*CS*_ = 400 nm [34,35] (see Table A in S1 Text) placed at the spindle poles’ locations. The MTs are elastic, semi-stiff filaments described using three interaction centers: the MT minus-end bead, the MT body bead, and the MT plus-end bead. These are 12-nm spherical beads, each representing ~40 αβ-tubulin dimers (size of αβ-tubulin dimers is ~4 nm). The MTs extend from the centrosomes in random directions. The MTs minus-ends are anchored by effective stiff angular springs on the surface of CSs. According to experiments, there are 200–3400 MTs per centrosome [12,36]; in the current implementation, 750 MTs per centrosome are in the model. The model also includes centromeres described as spherical particles with the radius *R*_*CH*_ = 362.5 nm [37] (Table A in S1 Text). Centromeres (‘naked CHs’) can be modeled with or without the CH arms. Centromeres and sister chromatids’ CH arms are represented by spherical beads with 362.5-nm radius; the contour length of the single CH is 6 μm, and each sister chromatid is represented by 8 beads, 4 beads extending on both sides of each KT/centromere region. The CH arms are capable of stretching and bending. The CH ends swing away from each other by a distance not to exceed 2 μm, which mimics the constraints on CH arms due to the presence of cohesin rings [38]. The KT surface is approximated by a cylindrical surface fragment [6] with the surface area *A*_*KT*_ = 0.15 μm^2^ (Table A in S1 Text) [39] (Fig 2C). The KT is modeled as a dense grid of small spherical particles of radius *R*_*Ndc*_ = 4 nm [40] (Table A in S1 Text) connected by elastic springs. A few of these KT particles are connected by springs to the inner KT center, and then the inner sister KT centers are interconnected by the centromere spring (Fig 2). The Ndc80 proteins associate with KTs [41–44], link the MT plus-ends with the KTs (see Table A in S1 Text), and allow the MT filaments to exert pulling forces in the range of a few to tens of picoNewtons on CHs [21,22,45–47]. We model Ndc80 proteins as elastic springs attached to the KT surface beads and capable of forming the linkages with MTs. A summary of all mechanically active components–MTs, sister KT pairs, CHs and Ndc80 binding domains, is provided in Table 1.

#### Biochemically active components

**Fig 4:**
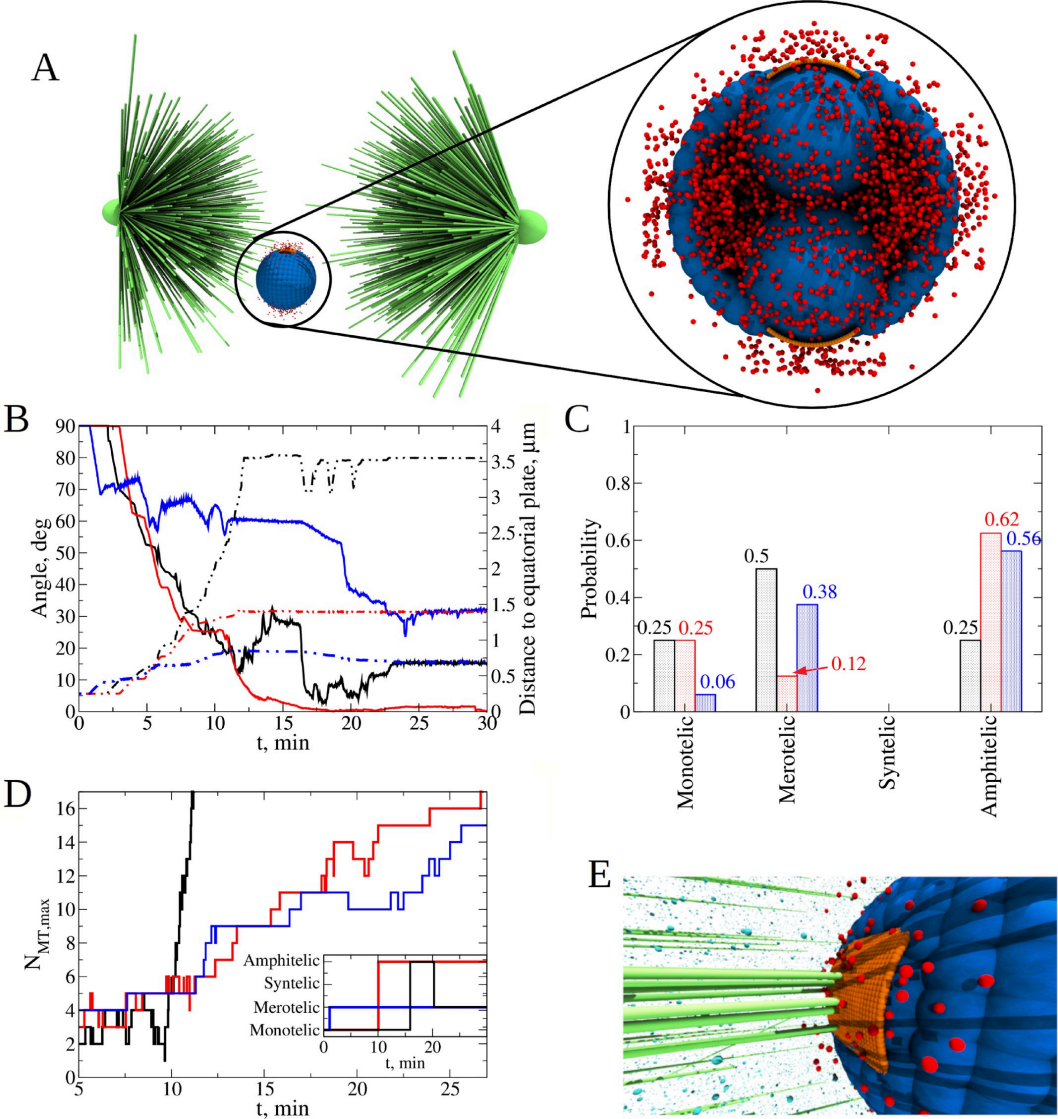
Exploring the role of Phosphatase and Aurora B kinase on dynamics of kinetochore-microtubule attachments. **A)** Initial position and orientation of the KT pair inside the cell. The KT pair is placed close to the equatorial plate, shifted by 1.5 μm below and along the axis perpendicular to the axis of the spindle. Orientation of the KT pair axis is along the axis perpendicular to the spindle axis. The AB particles are spatially distributed around the center between the KT pair as shown in the blowout. **B)** Time dependent evolution of KT-KT orientation angle (solid lines; left *y*-axis) and distance between KT-pair and equatorial plate (dash-dotted lines, right *y*-axis) for the Phosphatase to Aurora B (P:AB) ratio = 1:100 (blue), 1:10 (red), and 1:1 (black). Each curve shows results from a single simulation run. **C)** Probability to find each type of attachment for different P:AB ratio = 1:100 (blue bars), 1:10 (red bars), and 1:1 (black bars). Statistics was collected from *n* = 8 independent runs for each of these three cases. **D)** The P:AB ratio influences the average bond lifetime and the frequency of attachment-detachment switches. Shown is evolution of the maximum number of microtubules (MTs) attached to a single KT from a single centrosome for P:AB ratio = 1:100 (blue lines), 1:10 (red) and 1:1 (black) during 30 min of simulation. Changes in the KT-MT attachment status are shown in the *inset*. **E)** Snapshot of the final state of the system (red and cyan spheres are particles of Aurora B kinase and Phosphatase).

**Fig 5:**
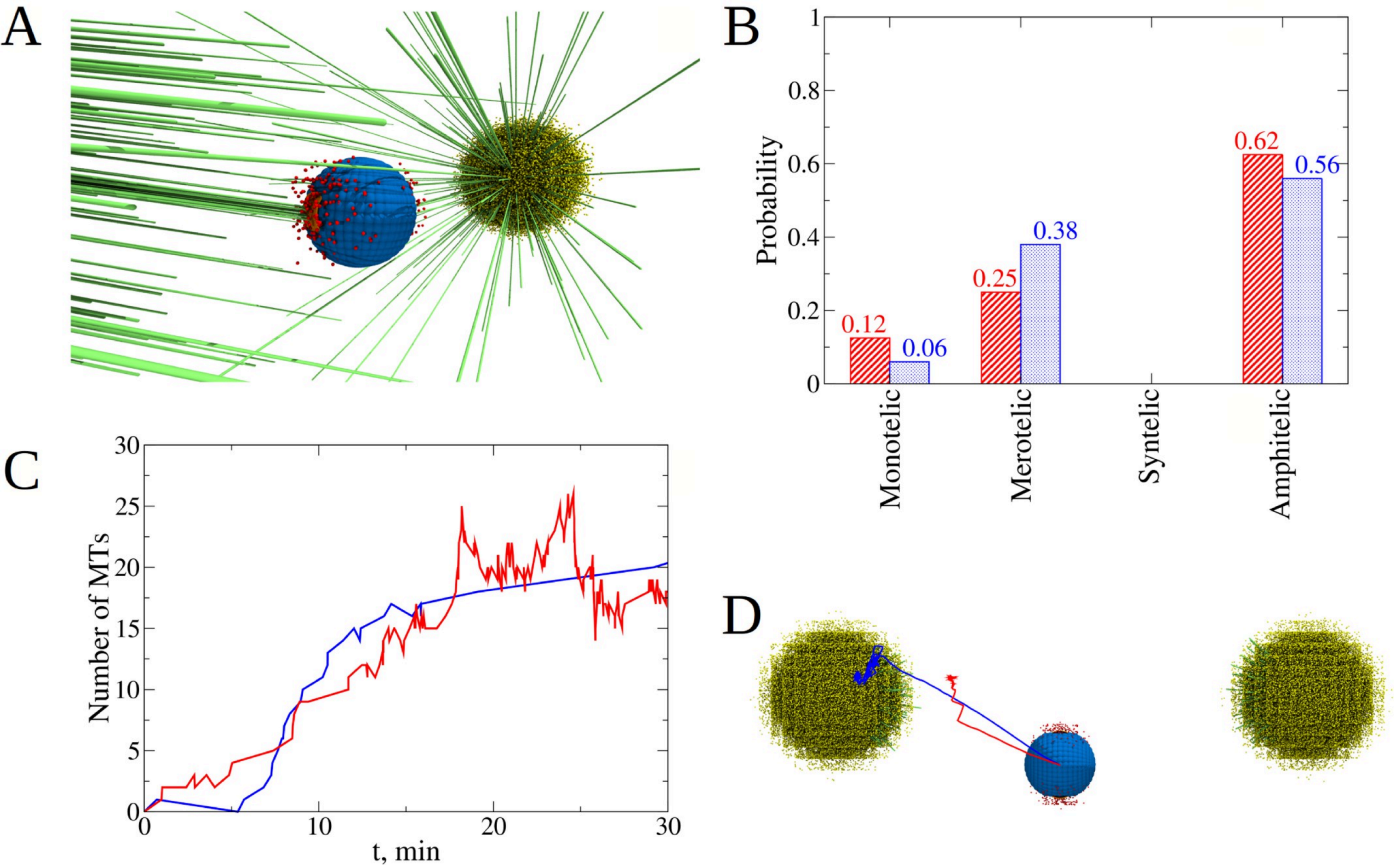
Exploring the effect of the Aurora A presence. **A)** Snapshot of the final KT pair position and orientation (amphitelic attachment) for a simulation with AA. **B)** Probability to find each type of attachment for the system without AA (blue bars) and for the system with AA (red bars). Statistics were collected from *n* = 20 simulation runs for both cases. **C**) Addition of AA kinases to the system changes the Phosphatase to Aurora B ratio close to centrosomes (CSs). This influences the average bond lifetime and the frequencies of attachment/detachment switches. Blue line shows the evolution of total number of microtubules (MTs) attached to a single KT pair from both CSs for the case study without AA, red line shows the number of MTs for the case study with AA. **D**) An example of how adding AA kinases to the system corrects the trajectory of the KT pair. Lines show the 2D projection (*xz*-plane) of the KT pair trajectory during 30 min simulations. For the case study without AA (blue line), KT pair reaches one of the CSs and for the case study with AA (red line), KT pair stops right before the cloud of AA kinases.

These are phosphatase (PH), Aurora A (AA) and Aurora B (AB) kinases, and MT-Ndc80 complex (see Table 2). These components are described using the RDME formalism. The binding affinity and, hence, the average lifetime of KT-MT attachments are modulated by the phosphorylation states of Ndc80. AA and AB phosphorylate the Ndc80 tails at up to 7 sites, whereas phosphatase dephosphorylates Ndc80 [48,49]. AA and AB undergo diffusion inside the cell. According to experiment [50], in the model phosphatase molecules are uniformly distributed in the interior space of the cell, and so we do not model their diffusion explicitly. Because AB is located in the area between the sister KTs [50], it is modeled implicitly by the spherically symmetric gradient of its concentration with the central maximum located in the space exactly in the middle between sister KTs (see Figs 1, 2, 4A and 5). AA is described by two spherically symmetric concentration gradients with the twin maxima centered on the two centrosomes (see Figs 1 and 5). The cell boundary (Fig 1) is modeled parametrically. One can select cell shapes to be either spherical, elliptic, cubic, or rectangular. Here, we choose the elliptic shape to describe the eukaryotic cell based on the experimental observations [6]. Fig 1 shows the interior space of the cell, which contains Phosphatase and Aurora A and B kinases. A summarized description of the biochemical components–MT-Ndc80 complex, PH, AA, and AB–is provided in Table 2.

#### Reaction-diffusion master equation formalism

We employed the RDME approach to modeling biochemical reactions and molecular transport [30,31,33]. The volume (~850 μm^3^) of the ellipsoidal shaped cell is divided into a large number of subvolume elements (subcells) of dimension *l*_*SV*_ = 250 nm (Table A in S1 Text), and the biomolecules in the cell are distributed among the subcells (Fig 1). Biochemical reactions are allowed only between molecules within a subcell. The biomolecules can diffuse randomly between the nearest-neighbor subcells. The state of the cell ***X*** is specified by the number of biomolecules and biomolecular complexes (e.g. KT-MT attachments) *x*_*j*,*v*_ of each type *j* = 1, 2, …, *J* in each subcell *ν* = 1, 2, …, *V*. The time evolution of the probability for the cell to be in state ***X*** is given by the sum of contributions from biochemical reactions, described by the reaction operator ***R***, and from molecular diffusion events, described by the diffusion operator ***D***, i.e.


$$\frac{dP(X,t)}{dt}=RP(X,t)+DP(X,t)=\sum_{v}^{V}\sum_{\mu }^{M}\left[\alpha_{\mu }(x_{v}-S_{\mu })P(x_{v}-S_{\mu },t)-\alpha_{\mu }(x_{v})P(x_{v},t)\right]+\sum_{v}^{V}\sum_{\xi }^{\pm i,j,k}\sum_{j}^{J}[d_{j}(x_{j,\nu +\xi }+1)P((x+{1)}_{j,\nu +\xi }-1_{j,\nu },t)-d_{j}x_{j,\nu }P(x_{j,\nu },t)]$$ (1)

In the second line in Eq 1, which describes the rate of change of *P*(***X***,*t*) due to biochemical reactions, ***x***_***v***_ is the column vector containing the number of molecules in the *ν*-th subcell, *α*_*μ*_(***x***_***v***_) is the reaction propensity for the *μ*-th reaction (*μ* = 1, 2,…, *M*) to occur in the *ν*-th subcell, and ***S***_***μ***_ is the *μ*-th column of the *J*×*M* stoichiometry matrix ***S***, which describes the changes in the number of molecules when the *μ*-th reaction occurs. In the absence of molecular transport, Eq 1 reduces to the spatially homogeneous chemical master equation (CME): $\frac{dP(X,t)}{dt}=\sum_{v}^{V}\sum_{\mu }^{M}\left[\alpha_{\mu }(x_{v}-S_{\mu })P(x_{v}-S_{\mu },t)-\alpha_{\mu }(x_{v})P(x_{v},t)\right]$, which is widely used to stochastically model chemical reactions in a well-stirred volume. Therefore, the RDME extends the CME to account for spatial degrees of freedom. In the third line in Eq 1, which describes the rate of change of *P*(***X***,*t*) due to molecular diffusion events, *d*_*j*_ is the diffusion propensity for a biomolecule of type *j* to move from subcell *v* to the next-neighbor subcell *v*+*ξ*, where *ξ* is a next-neighbor subcell in the ±*x*, ±*y*, and ±*z* direction (total of 6 next-neighbor subcells) denoted by the unit vectors ***i*,*j*,*k*** (see Fig A in S1 Text), *x*_*j*,*ν*_ is the number of biomolecules of type *j* in subcell *v*, and 1_*j*,*ν*_ represents a single molecule of type *j* in subcell *v*. In the absence of chemical kinetics, Eq 1 reduces to the diffusion equation, $\frac{dP(X,t)}{dt}=\sum_{v}^{V}\sum_{\xi }^{\pm i,j,k}\sum_{j}^{J}[d_{j}(x_{j,\nu +\xi }+1)P((x+{1)}_{j,\nu +\xi }-1_{j,\nu },t)-d_{j}x_{j,\nu }P(x_{j,\nu },t)]$.

The RDME (Eq 1) was sampled numerically using the Gillespie approach, which is based on the propensities, rather than probabilities of chemical reactions used in more traditional Monte Carlo approaches. In the Gillespie approach, the probability that the *μ*-th reaction will occur within the next time interval between *t*+*τ* and *t*+*τ*+*dt* is given by *P*_0_(*t*+*τ*)*c*_*μ*_*h*_*μ*_*dt*, where *P*_0_(*t*+*τ*) is the probability that at time *t*+*τ* no reaction has occurred in the previous time interval (*t*, *t*+*τ*). The reaction propensity for the *μ*-th reaction is given by *α*_*μ*_ = *c*_*μ*_*h*_*μ*_ and total propensity for all *M* reactions is $\alpha_{0}=\sum_{\mu =1}^{M}\alpha_{\mu }$. For a unimolecular reaction *μ* = 1, 2,…, *M* (e.g. MT-Ndc80 complex dissociation, MT growth, MT shortening, MT catastrophe and MT rescue; see Tables 3 and 4) to occur in the *ν*-th subcell with the rate constant *k* = *k*_*μ*_, the rate equation for a chemical species of type *A* is $\frac{dx_{A}}{dt}=-kx_{A}$. We define *c*_*μ*_*dt* = *cdt* to be the probability that a particular combination of reactants will interact through the same reaction *μ* in the time interval *dt*. If *h*_*uni*_ is the total number of distinct molecular reactant combinations at time *t*, then for a single molecule of type *A*, *c* = *k* and *h*_*uni*_ = *x*_*A*_, and the reaction propensity is *α*_*uni*_ = *ch*_*uni*_ = *kx*_*A*_. For a bimolecular reaction (e.g., Ndc80 phosphorylation, dephosphorylation and MT-Ndc80 complex formation; see Table 4), the rate equation for species *A* and *B* is $\frac{dx_{A}}{dt}=\frac{dx_{B}}{dt}=-kx_{A}x_{B}$. If *h*_*bi*_ is the number of distinct molecular reactant combinations for a bimolecular reaction at time *t*, then for a single combination of *A* and $B,c=k/l_{SV}^{3}$ (*l*_*SV*_ is the size of subcell) and *h*_*bi*_ = *x*_*A*_*x*_*B*_, and the reaction propensity is $\alpha_{bi}=ch_{bi}=kx_{A}x_{B}/l_{SV}^{3}$. The diffusion propensity for a molecule of type *j* (*j* = 1, 2, …, *J)* to move from subcell *v* to the next-neighbor subcell *v*+*ξ* is given by $d_{j}=\frac{D_{j}}{{l_{SV}}^{2}}$, where *D*_*j*_ is the diffusion constant (see Table 3 and Table A in S1 Text).

**Table 4:** Biochemical reactions at kinetochore-microtubule interface. Enzymatic reactions (e.g., phosphorylation and dephosphorylation) and association-dissociation reactions, which involve the MT associated protein Ndc80 linking MTs with KTs, and the reaction rate constants and characteristic timescales. The subscript *p* = 0, 1,…,6 denotes the number of phosphate groups attached to Ndc80 and changes in the rate constants and reaction propensities for MT-Ndc80 complex dissociation.

Chemical process	Reaction scheme	Rate constant *k*	Propensity *c*	Timescale
Ndc80 phosphorylation	${Ndc80}_{p}\to {Ndc80}_{p+1}$	1.5×10^7^ s^-1^ M^-1^	1.5 s^-1^	6.6×10^−1^ s
Ndc80 Dephosphorylation	${Ndc80}_{p+1}\to {Ndc80}_{p}$	3.0×10^7^ s^-1^ M^-1^	3.0 s^-1^	3.3×10^−1^ s
MT-Ndc80 complex formation	$MT+{Ndc80}_{p}\to MT:{Ndc80}_{p}$	3.8×10^9^ s^-1^M^-1^	3.8×10^2^ s^-1^	2.6×10^−3^ s
MT-Ndc80 complex dissociation	$MT:{Ndc80}_{p}\to MT+{Ndc80}_{p}$	(1.5+0.2*p*)×10^−3^ s^-1^	(1.5+0.2*p*)×10^−3^ s^-1^	5.7–11.1 min

The RDME formalism asymptotically approximates the Smoluchowski diffusion-limited reaction method when the average time for the next reaction to occur in a subcell *τ*_*R*_ is much longer than the average time until the next diffusion event *τ*_*D*_, i.e. *τ*_*R*_≫*τ*_*D*_ [30–32,51]. In the SRDDM, the slowest diffusing particles are Aurora A and B, for which the diffusion timescale is *τ*_*D*_ = 4.3×10^−4^ s (Table 3), and the most rapid reaction is MT-Ndc80 complex formation, for which the characteristic time is *τ*_*R*_ = 2.6×10^−3^ s (Table 4). Therefore, the diffusion timescale is ~10-fold shorter than the characteristic reaction time. This large separation of timescales for kinetics and diffusion (*τ*_*D*_≪*τ*_*R*_) justifies our using the RDME formalism. We adapted the multi-particle diffusion (MPD) method [52] and the next-subvolume method (NSM) [51,53] for exactly sampling the RDME (Eq 1).

### Force field for mechanically active components

#### Bead-spring representation

To describe the dynamic mechanical processes within the mitotic spindle assembly, we introduce mechanical energies and interaction forces between MTs, KTs, CHs, KT-MT attachments, and the cell boundary. The particles described by a single interaction center (bead) are centrosomes and KTs. Other components are represented by several (three or more) beads connected by harmonic springs. The sister KT pair is described by a pair of beads (two interaction centers) of radius *R*_*CH*_ = 362.5 nm connected by a harmonic spring with the spring constant *K*_*KT*,*r*_ = 3.3×10^3^ pN/nm (Fig 2A and 2B; see Table A in S1 Text). An MT is described by three beads, each of radius *R*_*MT*_ = 12 nm [54], linearly connected by the harmonic spring. Each MT filament is described by the stretching stiffness *K*_*MT*,*r*_ = 16.7 pN/nm and bending rigidity *K*_*MT*,*θ*_ = 7.7×10^5^ kJ/mol·rad^2^ (Table A in S1 Text). We describe the CH arms by using 5–9 beads of radius *R*_*CH*_ = 362.5 nm for each arm, depending on the 4–6 μm length of the arm (Table 1 and Table A in S1 Text), connected by harmonic springs. The flexible CH arms are described by the stretching stiffness *K*_*CH*,*r*_ = 3.3×10^3^ pN/nm, and bending rigidity *K*_*CH*,*θ*_ = 2.5×10^5^ kJ/mol·rad^2^ (Table A in S1 Text). The KT surface, modeled by a grid of particles of radius *R*_*Ndc*_ = 4 nm, is schematically illustrated in Fig 2A and 2B. There are 750 beads per corona surface (Table A in S1 Text) connected to each other by harmonic springs with the spring constant *K*_*Ndc*,*r*_ = 3.1×10^2^ pN/nm contained within the sphere of radius *R*_*Ndc−bond*_ = 200 nm. Selected beads in the KT surface are also connected to the KT center (virtual particle of radius *R*_*Ndc*_ = 4 nm) by a harmonic spring with the spring constant *K*_*KT*,*r*_ = 3.3×10^3^ pN/nm (Fig 2A and 2B; Table A in S1 Text). Since the KT surface is a biologically flexible structure, in CellDynaMo it may be varied between two 3D extremes, the flat (*χ* = 1) and maximally curved (*χ* = 0) versions of fixed area *A*_*KT*_ = 0.15 μm^2^ (Fig 2C and 2D; Table 1 and Table A in S1 Text). In all simulations presented here, we set *χ* = 0.5. We used the bead-spring representation to model the Ndc80 mediated KT-MT attachments. When an MT bumps into KT, Ndc80 forms a link between the plus-end of a growing MT (last bead of MT) and the closest bead on the KT surface within a sphere of radius *l*_*Ndc*_ = 65 nm [55,56], which is the length of Ndc80 (Table A in S1 Text). Ndc80 is modeled as a harmonic spring with the spring constant *K*_*Ndc*,*r*_ = 3.1×10^2^ pN/nm (Table A in S1 Text).

#### Mechanical energy

The mechanical energy for a cell configuration ***r*** is specified in terms of the positions of all the mechanically active components of the cell, ***r*** = ***r***_1_, ***r***_2_,…,***r***_*N*_. The total potential (mechanical) energy function for a cell configuration *U*(***r***) is given by the sum of potential energy terms for all mechanically active components and all the attachments:

$$U(r)=U_{MT}(r_{MT})+U_{CH}(r_{CH})+U_{KT}(r_{KT})+U_{att}(r_{MT},r_{KT})+\sum_{ij}U_{rep}(r_{ij})+\sum_{i}U_{mem}(r_{i})$$ (2)

In Eq 2, *U*_*MT*_, *U*_*CH*_, *U*_*KT*_, and *U*_*att*_ are the potential energies of all MTs, CHs, KTs, excluded volume interactions, and all KT-MT attachments, respectively; *U*_*rep*_ and *U*_*mem*_, represent excluded volume interactions between various components and the cell boundary.

*MT filaments*: Each MT is described by the stretching potential with the *i*-th and *j*-th bead-to-bead distance *r*_*MT*,*ij*_ and bending potential with the bending angle formed between the *i*-th, *j*-th and *k*-th bead, *θ*_*MT*,*ijk*_,

$$U_{MT}=U_{MT}^{str}+U_{MT}^{bend}=\sum_{ij}\frac{1}{2}K_{MT,r}{\left(r_{MT,ij}-r_{MT,0}\right)}^{2}+\sum_{ijk}\frac{1}{2}K_{MT,\theta }{\left(\theta_{MT,ijk}-\theta_{MT,0}\right)}^{2}$$ (3)

In Eq 3, *K*_*MT*,*r*_ and *K*_*MT*,*θ*_ are the stretching stiffness and bending rigidity for MT (Table A in S1 Text), *r*_*MT*,0_ is the equilibrium bead-to-bead distance which depends on the length of an MT filament, and *θ*_*MT*,0_ = 180° is the equilibrium bending angle (Table A in S1 Text).

*Sister KT pair*: A sister KT pair is described by the stretching potential with the bead-to-bead distance *r*_*KT*,*ij*_, stretching potential in the KT grid with the bead-to-bead distance *r*_*Ndc*,*ij*_, and stretching potential between KT and beads in the KT grid with the bead-to-bead distance *r*_*KT−Ndc*,*ij*_,

$$U_{KT}=U_{KT}^{str}+U_{Ndc}^{str}+U_{KT-Ndc}^{str}=\sum_{ij}\frac{1}{2}K_{KT,r}{\left(r_{KT,ij}-d_{KT}\right)}^{2}+\sum_{ij}\frac{1}{2}K_{KT,r}{\left(r_{Ndc,ij}-r_{Ndc,0}\right)}^{2}+\sum_{ij}\frac{1}{2}K_{KT,r}{\left(r_{KT-Ndc,ij}-r_{KT-Ndc,0}\right)}^{2}$$ (4)

In Eq 4, *K*_*KT*,*r*_ is the stretching stiffness for the sister KT pair and beads on the KT surface (Table A in S1 Text), and *d*_*KT*_ = 725 nm, *r*_*Ndc*,0_ = 8–200 nm and *r*_*KT−Ndc*,0_ = 362.5–400 nm are, respectively, the equilibrium distance between the sister KT beads (Table A in S1 Text), equilibrium distance between beads in the KT grid (beads have different values of *r*_*Ndc*,0_ because bonds between them are formed within the sphere of radius *R*_*Ndc−bond*_ = 200 nm; see Fig 2), and equilibrium distance between the beads in the KT grid and the center of KT (all beads have different *r*_*KT*−*Ndc*,0_ values; see Fig 2).

*CH arms*: Each flexible CH arm is described by the stretching potential with the bead-to-bead distance *r*_*CH*,*ij*_ and bending potentials with the bending angle *θ*_*CH*,*ijk*_ within a single CH, and stretching potential with the bead-to-bead distance *r*_*coh*,*ij*_ between the corresponding beads of the two sister CHs, (see Fig 1B, and Figs B and C in S1 Text),

$$U_{CH}=U_{CH}^{str}+U_{CH}^{bend}+U_{coh}^{str}=\sum_{ij}\frac{1}{2}K_{CH,r}{\left(r_{CH,ij}-r_{CH,0}\right)}^{2}+\sum_{ijk}\frac{1}{2}K_{CH,\theta }{\left(\theta_{CH,ijk}-\theta_{CH,0}\right)}^{2}+\sum_{ij}\frac{1}{2}K_{coh,r}{\left(r_{coh,ij}-r_{coh,0}\right)}^{2}$$ (5)

In Eq 5, *K*_*CH*,*r*_, *K*_*CH*,*θ*_ and *K*_*coh*,*r*_ are, respectively, the stretching stiffness and bending rigidity for CH arms, and stretching stiffness for sister CHs due to cohesin rings (see Table A in S1 Text); *r*_*CH*,0_ = 725 nm, *θ*_*CH*,0_ = 180° and *r*_*coh*,0_ = 725 nm are, respectively, the equilibrium bead-to-bead distance and equilibrium bending angle for beads within a single CH, and equilibrium bead-to-bead distance between corresponding beads in two sister CHs (Table A in S1 Text).

*KT-MT attachments*: We describe the KT-MT interactions (for the MT plus-end linked to KT by Ndc80) using the harmonic potential with the distance *r*_*MT−Ndc*,*ij*_ between a bead at the MT plus-end and a bead in the KT:

$$U_{att}=\sum_{ij}\frac{1}{2}K_{Ndc,r}{\left(r_{MT-Ndc,ij}-l_{Ndc}\right)}^{2}$$ (6)

In Eq 6, *K*_*Ndc*,*r*_ is the stretching stiffness of Ndc80 linker and *l*_*Ndc*_ = 65.0 nm is the equilibrium length of Ndc80 linker (Table A in S1 Text).

*Excluded volume interactions*: We describe the excluded volume interactions (between MTs and KTs, between MTs and CH arms, between KTs and CH arms, between CH arms of different CHs, and between different CHs) using the repulsive form of the Lennard-Jones potential with the inter-particle separation distance *r*_*ij*_:

$$U_{rep}=\sum_{ij}U_{ij}^{LJ}=\sum_{ij}\varepsilon {\left(\frac{\sigma }{r_{ij}}\right)}^{12}$$ (7)

In Eq 7, constant parameters *ε* and *σ* set the energy scale and length scale for excluded volume interactions, respectively; *σ* = *R*_*i*_+*R*_*j*_, where *R*_*i*_ and *R*_*j*_ are the radii of particles *i* and *j*, respectively, and *ε* = 2.1×10^5^ kJ/mol (see Table A in S1 Text).

*Cell boundary*: For the elliptical shape, function *ζ*_*i*_, determines whether the *i*-th mechanically active component (e.g. MTs, KTs and CHs) is inside the cell volume is given by

$$\zeta_{i}={\left(\frac{x_{i}}{a}\right)}^{2}+{\left(\frac{y_{i}}{b}\right)}^{2}+{\left(\frac{z_{i}}{c}\right)}^{2}$$ (8)

In Eq 8, *x*_*i*_, *y*_*i*_, *z*_*i*_ define the location of the center-of-mass of the *i*-th component and *a*, *b*, *c* are the semi-major and the two semi-minor axes of the ellipse, respectively (Table A in S1 Text). The potential energy due to soft harmonic repulsion with the component-boundary separation distance *r*_*i*_ is given by

$$U_{mem}=\sum_{i}\frac{1}{2}\Theta (\zeta_{i}-1)K_{mem}{r_{i}}^{2}$$ (9)

In Eq 9, *K*_*mem*_ = 3.3×10^3^ pN/nm is the boundary stiffness (see Table A in S1 Text) and *Θ*(*x*) is the Heaviside step function defined as *Θ* = 1 when *x* = *ζ*_*i*_−1> 0 and *Θ* = 0 otherwise.

### Langevin dynamics formalism describing mechanically active components

We used the Langevin Dynamics (LD) approach to model the dynamic mechanical processes. The cell configuration ***r*** is specified by the positions of all mechanically active components ***r***_*i*_, *i =* 1, 2,…, *N*, where *N* is the total number of components. The cell dynamic evolution was followed by integrating the Langevin equations in the overdamped limit for each position ***r***_*i*_ of each mechanically active component,

$$\frac{dr_{i}}{dt}=\frac{1}{\gamma }\frac{\partial U(r)}{\partial r_{i}}+\sigma g_{i}(t)$$ (10)

In Eq 10, U(***r***) is the total potential (mechanical) energy function (see Eq 2), *γ* is the friction coefficient, and ***g***_*i*_(*t*) is the Gaussian zero-average random force with the variance *σ*^2^. The Langevin equations were propagated forward in time with the timestep *δt* = 50 ps at room temperature (*T* = 300 K) using water viscosity (Table A in S1 Text). For the KT beads (*R*_*CH*_ = 362.5 nm, Table A in S1 Text), this corresponds to the friction coefficient *γ* = 6*πηR*_*CH*_ = 6.8×10^6^ pN ps/nm. For the MT beads (*R*_*MT*_ = 12 nm, Table A in S1 Text), this corresponds to the friction coefficient *γ* = 4.5×10^5^ pN ps/nm. The variance *σ*^2^ is related to the diffusion constant $D=\frac{k_{B}T}{\gamma }=\frac{\sigma^{2}}{2\gamma^{2}}$ (*k*_*B*_ is the Boltzmann’s constant), and so *σ*^2^ = 2*k*_*B*_*Tγ*. In Eq 10, $F_{i}=\frac{\partial U(r)}{\partial r_{i}}$ is the deterministic force and the second term contains the random force.

### Dynamics of microtubules: growth, shortening, catastrophe, and rescue

The MT growth is described by the equation $\frac{dl}{dt}=v_{gr}=k_{gr}\Delta l$ for the MT length *l*, where *k*_*gr*_ = 5.0 s^-1^ is the rate of growth and Δ*l* = 24 nm is the discrete increment of length. Similarly, the MT shortening is described using the equation $\frac{dl}{dt}=v_{sh}=-k_{sh}\Delta l$, where *k*_*sh*_ = 18.6 s^-1^ is the rate of shortening (Table 3 and Fig D in S1 Text). The reaction rate constants *k*_*gr*_ and *k*_*sh*_ are chosen to recover the experimental rates of MT growth and shortening, *v*_*gr*_ = 7.5 μm/min and *v*_*sh*_ = 27 μm/min (Table 3 and Table A in S1 Text). During the process of dynamic instability, MTs switch between phases of growth and shortening. The frequency of catastrophe *ω*_*cat*_ is set to the experimentally observed value [57], *ω*_*cat*_ = 2.5×10^−3^ s^-1^; the frequency of rescue *ω*_*res*_ is set to the experimentally observed value [57] *ω*_*res*_ = 3.0×10^−2^ s^-1^ (Table 3 and Table A in S1 Text).

### Dynamics of microtubules: interactions with chromosomes

In the SRDDM, MTs physically interact with CHs. These interactions are the following: 1) When an MT overlaps with a CH arm (or a centromere), the excluded volume interaction adds non-zero energy to the system (Eq 7) which generates a pushing force (the first term in the right-hand-side of Eq 10). The direction of this pushing force is along the line connecting the centers of two interacting spherical beads representing the MT and CH arm. This force is pushing the CH away from the MT, while also bending the MT away from the CH. As all MTs are either growing or shortening all the time, this excluded volume interaction lasts for a finite time interval. With the sizes of the beads and dynamic instability rates we use, the pushing process lasts for a short time rarely exceeding 2 s (see Fig E in S1 Text). After that, the MT effectively slides off the CH. The magnitude of the pushing force is determined by the parameter *ε* in the Lennard-Jones potential (Eq 7) chosen so that the average pushing force is in the 10-pN range (see Fig E in S1 Text) to conform with the experimentally established forces [46,58]. 2) When the plus-end bead of a growing MT is in the vicinity of a KT, which contains Ndc80 linker ends, the MT-Ndc80 bond formation takes place with the rate constant *k* = 3.8×10^9^ s^-1^M^-1^ (see Table 4). At the Ndc80 density used (~ 750 Ndc80 linkers per KT; Table 2 and Table A in S1 Text), this results in the formation of the Ndc80-MT attachment within, on average, 2.6×10^−3^ s (Table 4) as soon as the MT plus-ends bumps into the KT. 3) When an MT-Ndc80 attachment occurs, an immediate catastrophe takes place, and the MT starts to shorten. 4) During the KT-MT attachment time interval, the shortening MT stretches the Ndc80 spring thereby exerting the 10-pN average pulling force on the KT (see Fig E in S1 Text). The direction of the pulling force is along the line connecting the MT plus-end and the Ndc80 end bound to this plus-end.

### Numerical implementation

The SRDDM was mapped into CellDynaMo package (CUDA language), fully implemented on a GPU. In LD, the particle-particle interactions (e.g. excluded volume interactions, stretching and bending within the filament structures, such as MTs and CHs, and KT-MT attachment) are the computational bottleneck. However, these interactions are described by the same empirical potential energy function (Eqs 2–9). Therefore, when running Langevin Dynamics on a GPU, it is then possible to execute the same operation, e.g. generation of random forces, calculation of the potential energy, evaluation of forces, integration of Langevin equations of motion, for many particles at the same time. When mapping the RDME, we implemented the next-subvolume method (NSM) extension [51] of the original Gillespie algorithm [59,60]. In CellDynaMo, we implemented the multi-particle diffusion (MPD) approach to the reaction-diffusion master equation [52]. Numerical routines for the generation of (pseudo)random numbers (Hybrid Taus) for RDME and LD are described in our previous publications [61,62]. To achieve top performance on a GPU, all the numerical algorithms implemented in RDME and LD have been recast into a data-parallel form so that the computational threads run the same instruction stream, but on different data sets (i.e., subcells and particles). We made the tasks compute-intensive so that, most of the time, the GPU performs computations rather than reading and writing data. These efforts enabled us to reach the biologically important timescales. For example, it takes ~72 hours of wall-clock time to generate a few ~30 min trajectories of cell dynamics on a contemporary graphics card GeForce GTX 1080.

## Results

### Benchmark test simulations

To assess possible errors in the numerical implementation of the LD module for the mechanical components, and in the RDME description of chemical kinetics and molecular transport, we carried out benchmark test simulations. Here, we summarize the main results; technical details are given in S1 Text.

*Langevin Dynamics*: In CellDynaMo, the Langevin equations of motion in the overdamped limit for each position ***r***_*i*_ of each mechanically active component are integrated numerically using the first-order integration scheme (Ermack-MacCammon algorithm) [63]:

$$r_{i}(t+\delta t)=r_{i}(t)+\frac{1}{\gamma }\frac{\partial U(r)}{\partial r_{i}}\delta t+\sigma g_{i}(t)$$ (11)

First, to access the accuracy of the numerical integration, we carried out short 1-μs simulation runs (20,000 integration steps) for a small system of three beads interconnected by the harmonic springs (Fig F-A in S1 Text). Benchmark simulations of the cell dynamics have been carried out at zero temperature with the time step *δt* = 50 ps (see Table A in S1 Text). For this three-body system, the equations for the forces and displacements for all beads are known exactly (Eqs 1–3 in S1 Text), and so the results obtained both numerically and analytically can be directly compared. The displacements Δ*x*_13_ and Δ*x*_23_ displayed in Figs F-A, F-B in S1 Text shows excellent agreement between the exact and numerical results. The numerical error does not exceed 1.5% for *t*>1 μs. For a 5-pN force on bead 3, we obtained Δ*x*_13_ = 6.62×10^−1^ nm (simulations) vs. 6.66×10^−1^ nm (exact), and Δ*x*_23_ = 6.21×10^−1^ nm (simulations) vs. 6.27×10^−1^ nm (exact). For a 50-pN force, we obtained Δ*x*_13_ = 6.32 nm (simulations) vs. 6.33 nm (exact), and Δ*x*_23_ = 5.91 nm (simulations) vs. 6.33 nm (exact). We also investigated the dependence of the relative error $Err(\Delta x)=\frac{\left|{\Delta x}_{sim}-{\Delta x}_{exact}\right|}{{\Delta x}_{exact}}$ between the asymptotic values of the particle displacements obtained numerically (Δ*x*_*sim*_) and analytically (Δ*x*_*exact*_) for the 50-pN pulling force on the integration timestep *δt* = 5×10^−2^–5×10^2^ ps (see S1 Text for more detail). *Err*(Δ*x*) was found to be very low (<1.8%; see *the inset* to Fig F-B in S1 Text).

Next, we investigated the dependence of the relative error $Err(\langle \Delta x(t)\rangle )=\frac{\left|{\langle \Delta x(t)\rangle }_{sim}-{\langle \Delta x(t)\rangle }_{exact}\right|}{{\langle \Delta x(t)\rangle }_{exact}}$ between the average displacement for *N* = 100 Brownian oscillators obtained numerically (〈Δ*x*(*t*)〉_*sim*_) and exactly (〈Δ*x*(*t*)〉_*exact*_) on the cytoplasmic viscosity *η* = 1, 5, and 10 cPs and on the integration timestep *δt* = 5×10^−2^–5×10^2^ ps (see S1 Text for more detail). The numerical and analytical results practically collapse on the same curve (Fig F-C in S1 Text), and *Err*(〈Δ*x*(*t*)〉) was found to be very low, i.e. <0.4% for variable solution viscosity (*the inset* to Fig F-C in S1 Text) and <0.3% for variable timestep (Fig F-D in S1 Text; see *the inset* to Fig F-D in S1 Text for average relative error). Hence, our choice of *δt* = 50 ps as the timestep for Langevin Dynamics is reasonable.

We also carried out benchmark test simulations for a single KT pair (centromere; CH) to estimate the coefficients of one-dimensional translational diffusion and one-dimensional rotational diffusion based on the numerical output from the Langevin Dynamics simulations. These were then be compared with the exact analytical results for the translational diffusion coefficients, $D_{x}=\frac{k_{B}T∙ln\;(L_{CH}/d_{CH})}{4\pi \eta L_{CH}}$, and rotational diffusion coefficient, $D_{\theta }=\frac{k_{B}T∙3ln\;(L_{CH}/d_{CH})}{\pi \eta {L_{CH}}^{3}}$, of a cylinder (see Fig G in S1 Text). In these simulations, we set the CH height to *L*_*CH*_ = 4*R*_*CH*_ = 1.450 μm, and the CH diameter to *d*_*CH*_ = 2*R*_*CH*_ = 0.725 μm. We performed these test runs with a short timestep *δt* = 5 ps to obtain more detailed trajectories with a large number of KT positions and KT orientations (see Fig G in S1 Text). By averaging over 3 runs (each of 2.5-min duration), we obtained *D*_*x*,*sim*_ = 0.154 μm^2^/s (from simulations) vs. *D*_*x*_ = 0.152 μm^2^/s (exact result), and *D*_*θ*,*sim*_ = 0.866 rad^2^/s (from simulations) vs. *D*_*θ*_ = 0.869 rad^2^/s (exact result).

*Reaction-Diffusion Master Equation–Brownian diffusion*: We assessed the accuracy of the numerical implementation of the diffusion part of RDME by performing simulations of molecular transport, for which the exact distribution of the particles’ displacements is described by Gaussian statistics (Eq 4 in S1 Text). We carried out several short (100 s) benchmark simulations, in which we placed 10^4^ molecules of Aurora B in the central subcell *x*_0_ = 0 at time *t* = 0 (see Fig H in S1 Text). For Aurora B, we set the diffusion constant to *D* = 7.3×10^7^ nm^2^/s based on the Einstein-Stokes relationship for spherical particles (S1 Text) with the radius of *R*_*A*_ = 2.9 nm [64] (Table A in S1 Text). We ran the multi-particle diffusion algorithm (MPD; see Fig H in S1 Text) and observed spreading of molecules at later time points *t* = 1, 2, 5, 10, and 20 s, which correspond to 2.50×10^4^, 5.00×10^4^, 1.25×10^5^, 2.50×10^5^, and 5.00×10^5^ steps of iteration. The non-parametric density estimates of the distributions of particles’ displacements constructed based on the test runs are compared with the exact probability distribution curves in one dimension in Fig H in S1 Text, which shows excellent agreement between the numerical and exact results. We also compared the variance $\sigma_{x}^{2}$ from the simulations with the exact values $\sigma_{x}^{2}=2Dt$, for *t* = 1, 2, 5, 10, and 20 s. We found 1.4×10^8^ nm^2^ (simulations) vs. 1.5×10^8^ nm^2^ (exact) for the *t* = 1 s time point, 2.9×10^8^ nm^2^ (simulations) vs. 2.9×10^8^ nm^2^ (exact) for *t* = 2 s, 7.1×10^8^ nm^2^ (simulations) vs. 7.3×10^8^ nm^2^ (exact) for *t* = 5 s, 1.4×10^9^ nm^2^ (simulations) vs. 1.5×10^9^ nm^2^ (exact) for *t* = 10 s, and 2.8×10^9^ nm^2^ (simulations) vs. 2.9×10^9^ nm^2^ (exact) for *t* = 20 s. The numerical errors are all below 3%.

*Reaction-Diffusion Master Equation–Biochemical kinetics*: Next, we assessed the accuracy of the numerical implementation of the reaction part of the RDME. We carried out benchmark simulations for the consecutive two-step irreversible kinetics, $A\overset{k_{1}}{\to }B\overset{k_{2}}{\to }C$, with species *A*, *B* and *C* and reaction rate constants for the first and second steps, *k*_1_ and *k*_2_, and for the single-step reversible kinetics, *A*⥨*B*, with the reaction rate constants for the forward (*A*→*B*, *k*_1_) and backward (*B*→*A*, *k*_−1_) steps. For the two-step irreversible kinetics, the time-dependent populations *p*_*A*_(*t*), *p*_*B*_(*t*), and *p*_*C*_(*t*) are given by Eqs 8, 9, and 10 in S1 Text. For the single-step reversible kinetics, the populations *p*_*A*_(*t*) and *p*_*B*_(*t*) are given by Eqs 13 and 14 in S1 Text. We compared the results of numerical calculations of *p*_*A*_, *p*_*B*_, and *p*_*C*_ with the initial conditions *p*_*A*_(0) = 1, *p*_*B*_(0) = *p*_*C*_(0) = 0 and reaction rate constants *k*_1_ = 1 s^-1^ and *k*_2_ = 2 s^-1^ with the exact solutions given by Eqs 8, 9 and 10 in S1 Text. We compared the results of calculations of *p*_*A*_ and *p*_*B*_ with the initial conditions *p*_*A*_(0) = 1 and *p*_*B*_(0) = 0 and reaction rate constants *k*_1_ = 1 s^-1^ and *k*_−1_ = 3 s^-1^ with the exact solutions given by Eqs 13, 14 in S1 Text. The results are presented in Fig I in S1 Text, which shows that the time-dependent populations obtained analytically and numerically practically collapse onto the same curves.

### Optimal MT detachment rate maximizes correctness of KT-MT attachments

In the remainder of the paper, we apply our modeling toolkit to simulate the Search-and-Capture process for a single CH. The process of a single CH incorporation into the spindle is already so complex that in this paper we limit ourselves to this situation and leave the case of multiple CHs for the next study. Initially, there are two immobile CSs with dynamic MT asters and completely unconnected CH. We allow 30 min of biological time for MT and CH movements and attachment kinetics to play out, record the spindle angle (angle between the vector connecting sister KTs and the vector connecting the centrosomes/spindle poles), KT-KT distance, distance from the CH to the spindle equator, and number of MTs attached to KTs as functions of time. The results reveal complex and nuanced dynamics that results either in correct, amphitelic attachments, or, in a significant fraction of cases, in erroneous merotelic attachments or incomplete monotelic attachments. In a very few cases, the result is erroneous syntelic attachment. Analysis of the respective statistics gives unexpected insights about key feedbacks responsible for accuracy of the spindle assembly and pitfalls that the nascent spindle must circumvent.

We start with the ‘naked’ CH without chromosome arms. In this case, the CH is just a roughly spherical centromere with two sister KTs on the opposite sides of the centromere. We also start with the centromere spring to be very stiff, so that the KT-KT distance changes very little. This simplest case sheds light on one of the most basic questions–how the attachment accuracy depends on the rate of MT detachment from the KT. Previous modeling studies suggested that faster KT-MT detachment improves the accuracy, allowing destabilization of erroneous attachments. The main regulators of the KT-MT binding strength are members of the family of Aurora protein kinases and respective phosphatase enzymes. We model explicitly Aurora A (on the spindle poles) and Aurora B (on the centromeres), which reduce the KT-MT attachment strength by phosphorylation of the Ndc80 tails linking the MTs and KTs, and also the phosphatase enzymes, which make the KT-MT bond stronger via dephosphorylation of the Ndc80 tails. Therefore, it is only the number of Phosphatase and Aurora B (AB) molecules around the KT-MT interface that affect the turnover of individual KT-MT attachments. In the simulations, phosphatase, for simplicity, is uniformly distributed in the interior of the cell, while AB undergoes a confined diffusion and so is distributed with a spherically symmetric Gaussian distribution centered at the center of the centromere. The distribution width is such that AB spreads up to approximately 250 nm away from the centromere surface (see Fig 4A), where the 65-nm long Ndc80 linkers are located. The kinetic, force field and cell morphology parameters, including the Phosphatase-to-AB (P:AB) ratios, are given in Tables 1, 2, 3 and 4 and Table A in S1 Text.

To create an initial configuration, we placed the naked CH close to the equatorial plate, shifted it by 1.5 μm ‘downward’ along the axis perpendicular to the axis of the spindle, and oriented the KT-KT axis perpendicularly to the spindle axis (Fig 4A). This initial arrangement eliminates any positional and orientational bias, providing the same probability for MTs from both CSs to reach the same KT, which in turn should lead to a higher probability of forming merotelic attachments. In other words, these initial conditions, which we used in all simulations, are ‘making it hard’ for the CH to achieve the correct amphitelic state (see also S1 Movie).

Before we investigate how the Ndc80 phosphorylation state affects the KT-MT attachments, we first examine the effect of different P:AB ratios on the degree of Ndc80 phosphorylation *N*_*phos*_. We ran 3 sets of simulations for a single naked CH without Langevin Dynamics (LD is switched off), only with kinetics and diffusion (RDME is switched on), for 3 different P:AB ratios for 15 min of biological time. The number of AB enzymes was fixed at about 100, while the number of phosphatase molecules was varied from 10 to 100 and to 1000. Thus, the smallest P:AB ratio (P:AB = 1:10) results in the largest degree of phosphorylation, *N*_*phos*_ =6.3±0.7, which is close to the highest possible value (7 phosphorylated sites) that would result in the least stable KT-MT attachments. For larger P:AB ratios, *N*_*phos*_ = 3.3±0.4 (P:AB = 1:1) and *N*_*phos*_ = 0.1±0.0 (P:AB = 10:1), which should result in more stable KT-MT attachments.

Interestingly, the characteristic number of KT-MT attachments to a CH that remains close to the spindle equator (two of the three cases analyzed in Fig 4B, 4C and 4D) grows roughly linearly with similar rates despite different KT-MT detachment rates (Fig 4D). The reason is that roughly the same set of MTs growing into a sector that allows MTs to reach the CH, interact with the KTs. Thus, a detached MT often shortens, gets rescued, grows in roughly the same direction, and gets attached again. Note that after 30 min of biological time, the characteristic number of KT-MT attachments approaches 15 (Fig 4D and 4E), in agreement with the experimental observations in eukaryotes [65,66].

Eight independent simulation runs carried out for each P:AB ratio, provided the statistics shown in Fig 4C, which illustrates that slow KT-MT detachment leads to very inaccurate spindles–only 25% of amphitelic attachments evolve, while the majority, 50%, of the attachments are merotelic. The reason is that when a KT is exposed to both poles, there is a large probability for MTs from both poles to attach to the same KT, thus, creating the merotelic attachment. If the detachment rate is too slow, there is very little chance to break the erroneous attachments. Note that a surprisingly noticeable fraction of attachments, 25%, are monotelic. A close look at the CH trajectory, orientation and KT-MT attachment numbers (black curves in Fig 4B and 4D) hints that the origin of such attachments is the ‘geometric vicious cycle’; that is, if one of the KTs ‘acquires’ significantly more attachments to one pole than its sister KT to another pole, then the mechanical pulling starts to bring the CH closer to the first pole. The closer the CH is to the first pole, the more attachments the proximal KT accumulates, the stronger the pull to the first pole. The distal pole is becoming farther away from the CH, and the sister KT does not make enough attachments to the distal pole to stop this process. Sometimes, we observe that the sister KT does not have time to make even a single attachment from the distal pole. More often though, initially monotelic attachments turn into either amphitelic ones, when a MT from the distal pole reaches the unconnected KT (as happens around 15 min in the case shown in Fig 4D, black curves) or merotelic ones when an MT from the proximal pole reaches the KT previously unconnected to this pole (as happens around 20 min in the case shown in Fig 4D, black curves). Syntelic attachments were observed very rarely because rare transient syntelic connections rapidly turn into merotelic ones: in the syntelic state, when both sister KTs are attached to one pole, the CH is oriented so that both KTs are also exposed to the opposite pole causing rapid merotelic connection.

Simulation with an intermediate detachment rate revealed a dramatic improvement in attachment accuracy: while the fraction of the monotelic connections, 25%, is unchanged, the percentage of the amphitelic connections, 62%, increased more than two-fold, while the percentage of the merotelic connections, 12%, decreased four-fold. The reason is that many merotelic connections transiently lose a minor number of ‘incorrect’ MTs connecting one of the KTs to the ‘wrong’ pole. When this happens, the ‘beneficial’ geometric feedback kicks in: transient amphitelic connections, by pulling sister KTs to the opposite poles, orient the CH so that the spindle angle approaches zero, which enables the centromere body to protect each of the KTs from the ‘wrong’ pole and accelerates formation of attachments from the ‘right’ pole. Thus, the correct attachments improve the spindle angle, while better angles promote accurate attachments. The geometric vicious cycle does not occur because amphitelic attachments do not allow the CH to deviate from the spindle equator too much (see also S1 Movie).

Interestingly, when we explored the fastest KT-MT detachment rate, the accuracy worsened: though the number of amphitelic attachments decreased just a little, from 62% to 56%, the number of merotelic attachments increased significantly, from 12% to 38%. The reason is that too rapid MT attachment dynamics does not stabilize the amphitelic state, allowing frequently detaching CHs to rotate slightly into unfavorable positions exposing the KTs to wrong poles and causing merotelic attachments. Note also the drastic decrease in the number of monotelic attachments in this case. The reason is that the rapid dynamics, while ruining the beneficial geometric feedback, also disables the geometric vicious cycle: when one of the KTs starts moving toward one of the poles, the number of MTs from that pole pulling that KT does not grow fast enough due to the rapid detachment, and the movement slows down giving time for MTs from the opposite pole to attach to the sister KT and to stop the divergence from the equator. In summary, we conclude from this case study that there is an optimal intermediate KT-MT attachment rate maximizing the spindle accuracy.

### Aurora A kinase has a small positive effect on the attachment accuracy

Next, we investigated the influence of Aurora A kinase (AA) in the vicinity of the spindle poles (Fig 5A), which was switched off in the simulations for Fig 4 focused on the accuracy of KT-MT attachments. This case study was motivated by the possibility that AA could help in breaking the KT-MT attachments responsible for bringing the CH too close to one of the spindle poles. The idea is that near one of the poles, AA phosphorylating activity would increase the KT-MT detachment rate, which in turn would allow the CH to move further away from the pole and back to the center of the spindle, where the sister KT could then acquire proper KT-MT connections.

We carried out 20 independent simulations for the system without AA and then ran 20 simulations with AA present, whose Gaussian distributed concentration profile is centered on both poles (see Fig 5A and 5D), for 30 min of biological time. In these simulations, we used the highest P:AB ratio for AB, corresponding to the fastest KT-MT detachment kinetics; all the kinetics, mechanical and geometric parameters are given in Tables 1, 2, 3 and 4 and Table A in S1 Text. The addition of AA improved the accuracy (Fig 5B) but only slightly: the probability of formation of amphitelic attachments slightly increases, while the probability of formation of merotelic attachments decreases (Fig 5B). The AA effect on the total number of MTs attached to the naked CH can be seen in Fig 5C. In the absence of AA, the number of attached MTs starts to grow abruptly (a signature of the geometric vicious cycle) and changes little after the CH approaches one of the poles. In the presence of AA, the number of attached MTs grows more evenly, and, importantly, fluctuates significantly near the maximum, because AA frequently breaks the attachments to the proximal pole. As a result, occasionally, the effect of AA stopped the movement of the naked CH to one of the poles (Fig 5D). Equivalent behavior in the simulations without AA was not observed. In summary, AA does have a small but overall positive effect on correcting the KT-MT attachments and reversing incorrect motion of KT pairs toward the CS poles.

### Chromosome arms and centromere softening improve the accuracy of the spindle assembly

For the purpose of simplicity, in our previous simulations (Figs 4 and 5), we used a stiff centromere without the CH arms. Here, we can use those simulations as benchmarks to examine how the addition of the CH arms and softening of the centromere affects the number and distribution of KT-MT attachment types and the final orientation of the CH. The mechanical effect of the soft centromere is hypothesized to be important for stabilizing the amphitelic attachments: when sister KTs are pulled in the opposite directions by amphitelic attachments, the sister KTs deform the centromere and shift away from the ‘Aurora B cloud’. This decreases the degree of phosphorylation of the Ndc80 linkers, decreases the KT-MT detachment rates and stabilizes the connections. The erroneous connections either do not generate the stretch (syntelic) or generate a smaller stretch (merotelic) on CH (Fig J-E in S1 Text), and the detachments are faster, which increases the probability of a correction. It is impossible to simply intuit what the effects of the CH arms on the accuracy are, and this is where the detailed 3D simulations become crucial.

**Fig 6:**
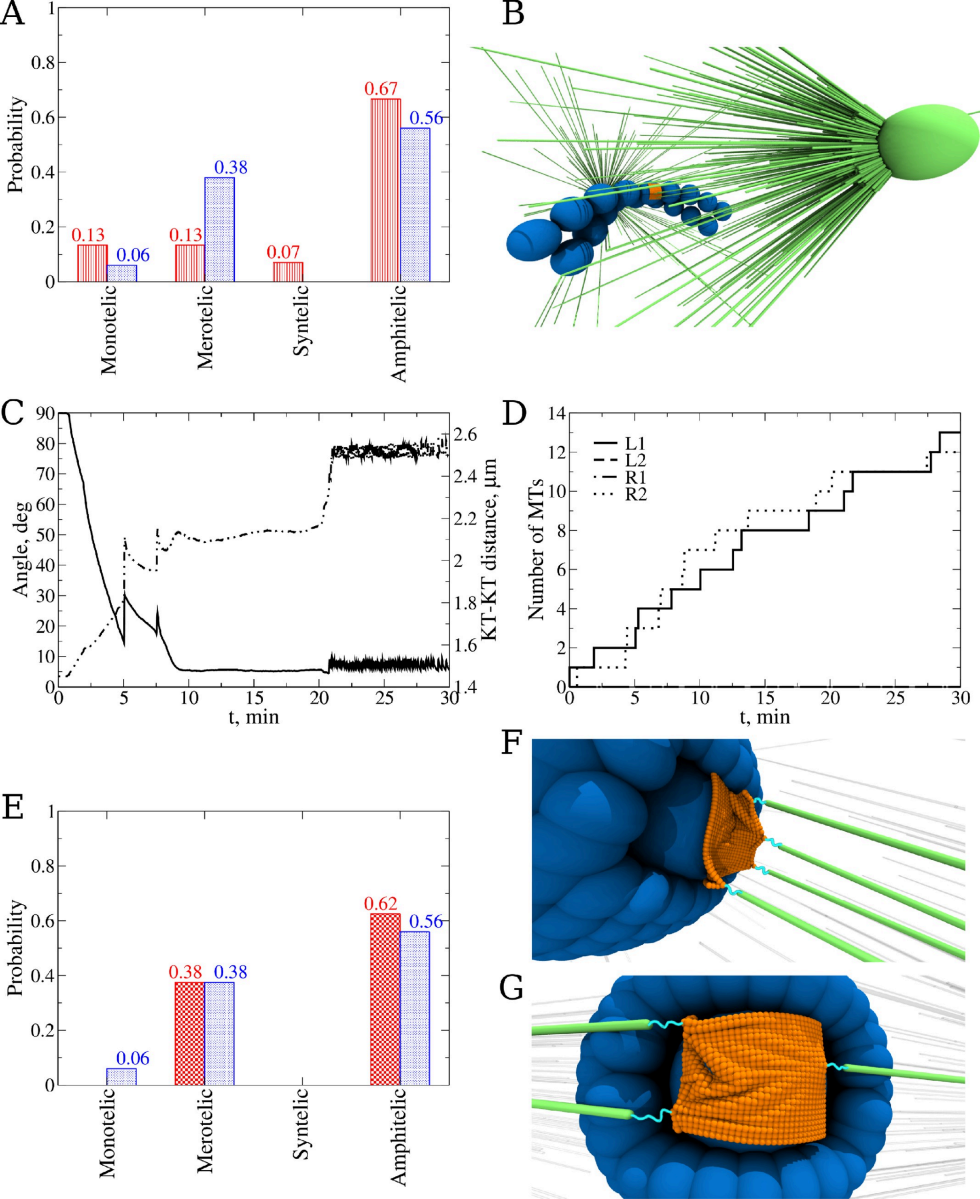
Describing chromosome arms and flexible corona surface in Stochastic Reaction-Diffusion-Dynamics Model. **A)** Probability to find each type of attachment for a single chromosome (CH) with CH arms. Blue and red bars represent statistics for kinetochore pairs (KTs) without and with CH arms, respectively. The statistics are based on *n* = 20 simulation runs for each case. **B**) Snapshot after 30 min simulation of biological time shows a representative example of merotelic attachment. **C)** Changes in KT-KT orientation angle (solid lines; left *y*-axis) and distance between KT-pair and equatorial plate (dash-dotted lines, right *y*-axis) over time for the most representative simulation run for the case study with CH arms present. **D)** Number of MTs vs. time profiles for the same simulation run as in panel **C** showing proper amphitelic attachment. Abbreviations used: L1 and L2 denote the numbers of MTs from the left CS attached to the first KT and second KT, respectively; R1 and R2 are the numbers of MTs from the right CS attached to the first KT and second KTs, respectively. **E)** Probability to find each type of attachment for a rigid corona surface (blue) and for a flexible corona surface (red). The statistics are based on *n* = 20 simulation runs for each case study. **F)** KT-MT interface and corona flexing for a case of amphitelic attachment. **G**) KT-MT interface and corona flexing for a case of merotelic attachment.

We carried out 2 sets of simulations for 30 min of biological time, In the first set, we ran 20 independent simulation runs for the naked CH with stiff centromere, and in the second set we then repeated 20 simulation runs with an entire CH (centromere plus CH arms) and stiff centromere. All parameters are given in Tables 1, 2, 3 and 4 and Table A in S1 Text. Initially, we positioned the centromere as described above (see Fig 4A; see also S2 Movie) with straight CH arms perpendicular to the spindle axis and the path from the poles to the KTs. Indeed, the accuracy improved with the addition of CH arms and with softening the centromere (Fig 6A). The characteristic dynamics of one example of an optimal path to amphitelic attachment is shown in Fig 6C and 6D. There was never an erroneous attachment in this case study. The spindle angle rapidly turned to zero, the number of MTs attaching to the KTs increased steadily and equally for both KTs, thus increasing the stretching force and pulling the KTs apart (see Fig J-E in S1 Text). One of the reasons for this improved accuracy is the soft centromere spring, but we also expect that since CH arms are large structures, they might slow the CH movement. This would keep the CH near the spindle center (see Fig 6B), where there is a higher chance of formation of correct attachments from both poles. We will confirm this intuition in the case studies described below.

### Small KT deformations do not significantly affect the accuracy of spindle assembly

The KT outer layer is likely not rigid but flexible, and it was hypothesized that this flexibility could play a positive role in correction of merotelic attachments [6]. If a ‘wrong’ MT connects to an otherwise amphitelically attached KT, this KT is already pulled by multiple amphitelic connections away from the AB cloud. However, the merotelic MT could then deform a part of the KT back into the AB cloud, which would accelerate the detachment of this MT. Therefore, we examined whether there was any effect of the KT flexibility on the number and types of KT-MT attachments. For this case study, we carried out simulations of a naked CH without CH arms. All parameters are given in Tables 1, 2, 3 and 4 and Table A in S1 Text. In this case study, we compared the results obtained for a rigid KT with the results for a soft KT. To model a soft KT, we decreased 40-fold the value of *K*_*KT*,*r*_ in Eq 4 for the potential energy $U_{KT-Ndc}^{str}$ going from a value of *K*_*KT*,*r*_ = 3.3×10^3^ pN/nm (rigid KT) to 82.5 pN/nm (soft KT). Also, we changed the cut-off radius *R*_*Ndc−bond*_ for the KT structure (with *χ* = 0.5 curvature) by decreasing it from 200 nm to 50 nm (see Fig 2A and 2B).

We carried out 20 independent simulation runs for 30 min of biological time both for rigid and deformable KTs. The results obtained for final KT-MT attachments observed at the end of simulations resulted in the statistics of KT-MT attachments shown in Fig 6E. We see that there is little difference between the results obtained for the rigid KT versus deformable KT. The reason is likely that the deformations brought about by forces exerted by the attached individual MTs are not large enough to exert the desired effect (see Fig 6F and 6G for the amphitelic and merotelic cases; see also S3 Movie for the amphitelic case).

### Thermal noise worsens accuracy of KT-MT attachments for naked centromeres but improves accuracy of CH possessing CH arms

**Fig 7:**
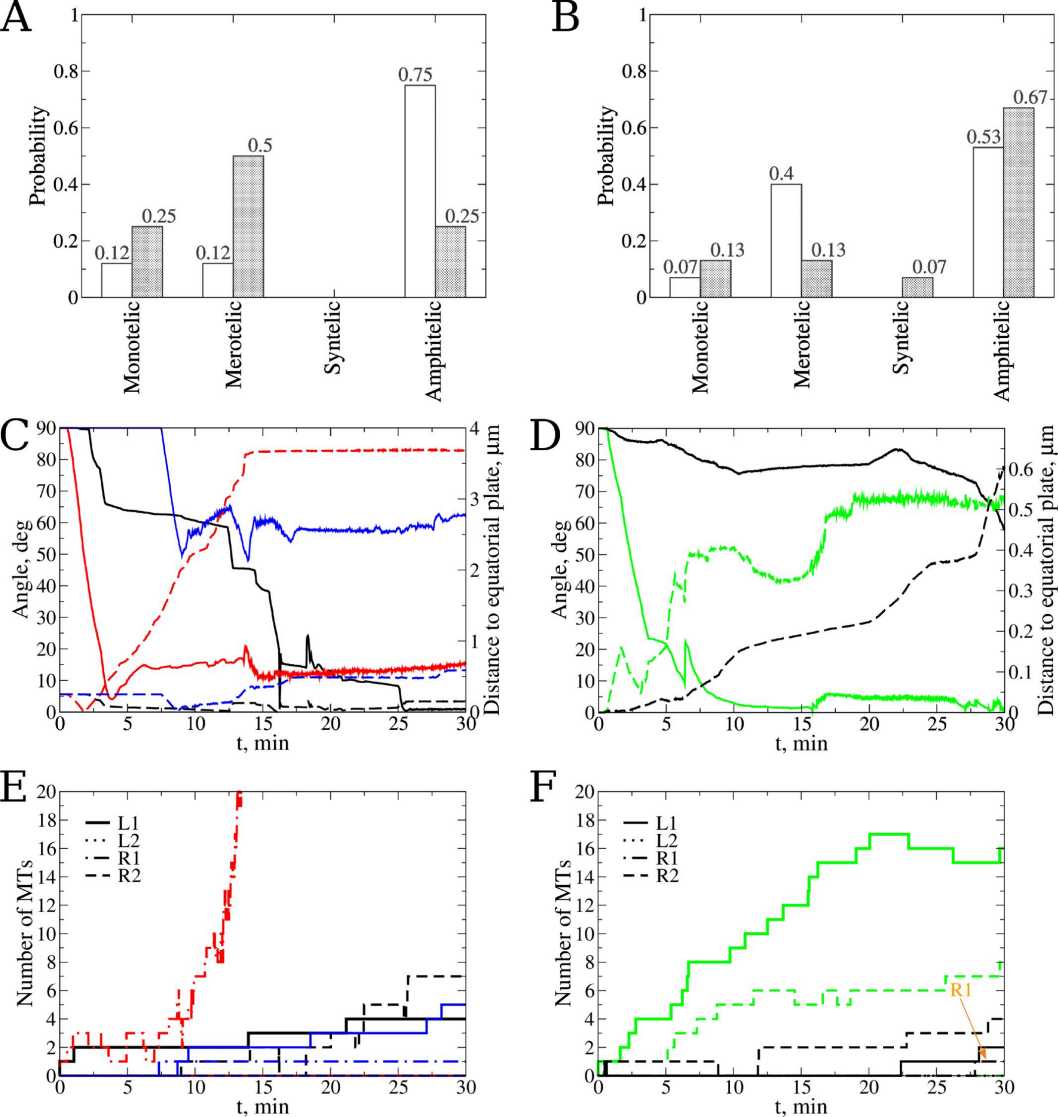
Influence of stochastic noise on dynamics of KT-MT attachments. Comparison of the types and numbers of KT-MT attachment from the simulations with the random force component (shaded bars) and without random force component (blank bars) for: **A**) A single KT pair with the Phosphatase to Aurora B (P:AB) ratio = 1:10, **B**) A single CH with CH arms (see also Fig J in S1 Text). The KT-KT orientation angle vs. time (solid lines) and distance to the equatorial plate vs. time profiles (dashed lines) for the case of a single KT pair with P:AB ratio = 1:10 (panel **C**) and for a single CH with CH arms (panel **D**). Black lines in panel **C** corresponds to simulation run without thermal fluctuations and demonstrate amphitelic attachment. Red and blue lines correspond to simulation runs with thermal fluctuations and demonstrate monotelic and merotelic attachments, respectively. In panel **D**, black and green lines represent simulation runs without and with thermal fluctuations, respectively. Panels **E** and **F** demonstrate the profiles for the number of MTs attached to KTs from both CS for a single KT pair with P:AB ratio = 1:10 and for a single CH with CH arms, respectively. In panels **E** and **F**, the assignment of curve color is same as in panels **C** and **D**. Abbreviation L1, L2, R1, and R2 are same as in Fig 6D.

Thermal noise often plays important roles in cells, helping to achieve robust biological functions [67–75]. For example, higher levels of stochastic noise result in the increased robustness of cell polarization [76]. Previous research has not addressed the role of thermal noise in mitosis, and so here we explored this role computationally by switching on and off the random force exerted on each mechanically active component. In all case studies reported above, the random force modeling stochastic cell environment (*σ****g***_***i***_(*t*) term in Eq 10) was switched on. For this case study, we performed independent simulations, which include: i) 8 trajectories for a naked CH with P:AB ratio = 1:100, 8 trajectories for a naked CH with P:AB ratio = 1:10, and 8 trajectories for a naked CH with P:AB ratio = 1:1; ii) 20 trajectories for a single naked CH with AA present; iii) 20 trajectories for a single CH with CH arms; and iv) 20 trajectories for a naked CH with flexible KT. The results of simulations with and without thermal noise are displayed in Fig 7.

Two sets of simulations show significant effects of noise. Specifically, for the case of a naked CH pair with P:AB ratio = 1:10, neglecting the random force component dramatically increases (by 3-fold) the number of amphitelic attachments, while the numbers of merotelic and monotelic attachments decreases 4-fold and 2-fold, respectively. The hint to the reason for this effect came from characteristic dynamics of the monotelic and merotelic attachments (red and blue curves in Fig 7C and 7E, respectively). Let us start with the monotelic ones: for the first ~7 min, a small number of MTs from the ‘right’ pole attach to the ‘upper’ KT (the one ‘exposed’ to both poles). In the first 3 min, these MTs pull rapidly turning the CH, exposing the attached KT further to the right pole and exposing it to more MTs from this pole. Starting from 2 min, these MTs start to rapidly pull the CH away from the equator and closer to the right pole. This proximity and beneficial angle create an avalanche of KT-MT binding between 7 and 15 min (Fig 7E), and by 15 min the CH practically falls on the right pole. (Fig 7A). In the case of the merotelic attachment (blue curves in Fig 7C and 7E) the following events take place: almost instantly, one MT from the right pole and another from the left pole bind to the upper KT exposed to both poles. The MTs pull in the opposite directions, which keep the CH near the equator and not rotated. This keeps the sister KT hidden, and it never makes any KT-MT attachments. Although the number of KT-MT attachments for the opposite poles becomes different, this difference does not significantly change the angle and position of CH.

Next, we compared the movements of the CHs and MTs with and without thermal noise. We found the following: CHs are large, and thermal forces have virtually no effect on them. However, the MTs are thin elastic rods, and thermal forces are capable of bending the MTs, and so the latter start to undergo rapid small-amplitude undulations. What turned out to be important is rapid trembling of the MT plus-ends. It enables the plus-ends to rapidly find a KT when a growing MT passes by a KT. Thus, effectively, this increases the KT-MT attachment rate. At first glance, this is beneficial for rapid assembly. However, this is not beneficial for accuracy! Indeed, either numerous MTs rapidly attach to the upper KT from both poles, while the lower one is hidden, and then the CH never turns, thus keeping the lower KT hidden, or the first MT has time to rapidly turn the CH toward one of the poles, and then the geometric vicious cycle begins pulling the CH closer and closer to that pole and acquiring more and more MTs from that pole ensues. Of course, other scenarios exist (and were observed in the simulations), but these two cases illustrate the fundamental issue.

What improves the situation when the thermal noise is turned off is revealed by the amphitelic dynamics (shown in black in Fig 7C and 7E). The MTs do not tremble, there is a slower accumulation of attachments. Because of that, there is a sizable time interval between consecutive attachments, and the first attachment formed earlier has time to turn the CH before the second attachment from the opposite pole formed later in time preventing the CH turning. This CH turning makes the attached KT less visible to the opposite pole, while the sister KT is now more visible to the opposite pole, and so it is more likely to create an amphitelic attachment.

The surprise was that when we performed a comparison for the case of a CH with CH arms, the result was quite the opposite: thermal noise improved the accuracy. The probability of amphitelic attachment slightly decreased and the probability of merotelic attachment strongly increased (see Fig 7B). Dynamics shown in Fig 7D and 7F gives us a hint why this is the case. Green curves show the amphitelic attachment dynamics for the case with thermal noise. One can see that in this case the number of KT-MT attachments grows rapidly, to the correct poles. Indeed, the attachment rate is high because of the noise effect. The numbers of attachments are also asymmetric–the upper KT attaches to the left pole first, which rapidly turns the centromere, *but does not rapidly move the CH*. The reason is that due to CH size, it’s effective viscous drag is large. This prevents the geometric vicious cycle from starting. While the CH starts moving away from the equator very slowly, the rapid turn (turning of the small centromere is rapid, which can be seen from the movies; see S2 Movie) allows MTs from the opposite pole to attach to the sister KT, thus establishing the amphitelic connection. The characteristic merotelic connection in the case study without thermal noise is shown by black curves in Fig 7D and 7F. The MTs attach rarely (low attachment rate), CH does not shift much, and in the end the fatal merotelic attachment is formed.

The other two sets of simulations, i.e. with flexible KT and with AA, did not show significant differences between the simulations with and without thermal noise (Fig J in S1 Text).

## Discussion

### 3D model of search-and-capture process

We developed and tested the molecularly detailed, mechanochemical 3D Stochastic Reaction-Diffusion-Dynamics model (SRDDM) of the Search-and-Capture pathway of mitotic spindle assembly. This model extends beyond the previous modeling attempts [28] by taking into account realistic 3D geometry of CHs with deformable sister KTs of realistic size and shapes the flexible centromeric region, and elastic dynamic MTs. The model also simulates the diffusion of Aurora A and B kinase and incorporates the molecularly explicit kinetics of (de)phosphorylation of Ndc80 linkers, and resulting formation and rupture of Ndc80-mediated KT-MT connections. The SRDDM is based on solving the spatially inhomogeneous RDME for describing biochemical reactions and molecular transport, and LD to treat the force-dependent dynamic mechanical processes (Fig 3). The model combines the stochastic description of kinetic processes (enzyme catalysis, formation and rupture of protein-protein complexes) and molecular transport of chemical species in the cell interior (diffusion of Aurora A and B kinases and Phosphatase) with the stochastic description of force-generating processes, (CH pushing/pulling by growing/shortening MTs).

Several frameworks have been proposed for computational modeling of the cytoskeleton, and we briefly discuss the most relevant. One of the first computational platforms for numerical modeling of the cytoskeleton network was Cytosim [77], which has been very successful and widely used by the scientific community. This model describes the physics using Brownian dynamics and allows one to simulate interactions between flexible fibers and motors. Cytosim has been utilized to model a wide variety of cytoskeletal phenomena, including spindle dynamics. The main thrust of CellDynaMo is to integrate transport and biochemical reaction processes with cytoskeletal mechanics in 3D and to introduce more complex and molecularly detailed heterogeneity into modeling the intracellular environment, i.e. geometrically and mechanically realistic chromosomes and kinetochores. This builds on the Cytosim success and extends it further. An example of a more recent modeling environment is MEDYAN [78], a sophisticated model of the actomyosin network that includes a large number of molecular components and the cell membrane. As in CellDynaMo, in MEDYAN Langevin Dynamics is used to describe the force-dependent dynamic mechanical processes, and the Gillespie algorithm is implemented to treat the biochemical kinetics. MEDYAN is capable of simulating biological systems with intricate membrane-cytoskeleton interactions [79]. In Lattice Microbes [52], as in CellDynaMo, the next-subvolume extension [51] of the Gillespie algorithm [59,60] is used to sample the RDME. Odell and Foe developed a detailed 3D model of the spindle-related assemblies [23] based on a combination of mechanical interactions and transport equations. Another model is aLENS developed by Yan et al. [80]. This 3D model describes cytoskeletal assemblies and also includes MTs and crosslinkers. The aLENS employs the Brownian motion of MT filaments (with excluded volume interactions) and the kinetics of binding and dissociation of crosslinkers (Kinesin-5 and dynein motor proteins) described with the Monte Carlo algorithm. The aLENS model takes advantage of parallel simulations with MPI and OpenMP. Yet another cytoskeleton model is CyLaKS developed by Fiorenza et al. [81]. These authors also use the kinetic Monte Carlo algorithm to describe binding of motors and crosslinkers (kinesin-1 and PRC1) to MT filaments and Brownian Dynamics to update the filaments’ positions. In the CyLaKS model, the mechanochemistry (cross-communication between the kinetic and dynamic components) is realized as in CellDynaMo. A model of the cytoskeletal network developed by Belmonte et al. [82] uses the 2D Brownian Dynamics of MT filaments, and stochastic kinetics to account for binding and dissociation of molecular motors (myosins, kinesins, and dyneins) to and from the MT filaments.

### CellDynaMo package

We mapped the SRDDM into the CellDynaMo package. The initial input for computer simulations with CellDynaMo is provided by a user in a set of configuration files, which specify the kinetic parameters, e.g. reaction rate constants, number of copies, the force field parameters, e.g. stretching and bending rigidities of MTs and CH arms, strength of KT-MT attachments, and the cell morphology parameters, e.g. number of chromosomes, number of kinetochore pairs, membrane shape, etc. These parameters specify the geometry of spatial arrangements of various cell components (Fig 1), biochemical kinetics (Tables 2 and 3 and 4), diffusion of biomolecular species (Table 3), force-generating properties, and mechanical interactions (Table 1) for cell components. For example, in the configuration file for cell morphology one can describe the cell geometry (shape and size), the number of chromosomes, the length of chromosome arms, the shape and size of kinetochore corona surface, the number of microtubules per centrosome, the initial configuration of all cell components, and the boundary conditions (cell membrane). Information about the viscosity and temperature of the cell cytoplasm is contained in the distribution of random forces. In the kinetics configuration file, the user can specify the reaction network, copy numbers of biomolecules, and kinetic rate constants for all biochemical reactions. In the mechanics configuration file, the user can specify the properties for all force-generating components, including stretching and bending rigidities, strength of KT-MT attachment, etc. A full list of parameters used in CellDynaMo to specify cell morphology, and to solve numerically the RDME and LD equations is provided in Table A in S1 Text.

Numerical algorithms for sampling RDME and LD are organized into the following workflow. For each molecular component in every subcell, the RDME module determines if the component diffuses out of that subcell, records what neighboring subcell it diffuses into, and performs this check for all components. Next, for each subcell the RDME module checks if any reaction occurs, performs this check for all reactions and updates the system state. Biological time is determined by the number of steps of the RDME algorithm. Next, the algorithm checks if there are changes in the chemical state (see Table 4) that might lead to changes in the mechanical state (Fig 3). The mechanochemical coupling is realized through the particle-particle interactions (e.g. KT-MT attachment/detachment). When the reaction and diffusion events lead to changes in the mechanical state (e.g. formation/dissociation of KT-MT bond, MT pulling or pushing on chromosomes), the RDME module switches off. At this time point, the LD switches on and brings the cell to a new state of mechanical equilibrium. For example, if a dissociation of an MT from the KT occurs, this information is passed to the LD module which switches off the excluded volume interactions. When a new state of mechanical equilibrium is reached, the LD module switches off, and the RDME module is back on (similar approach is implemented in MEDYAN [78]). CellDynaMo generates a simulation output, which includes coordinate and force files for mechanically active components, and file with subcell specific content (e.g., copy numbers of chemical species in each subcell).

The results of application of CellDynaMo show that contemporary GPUs can be utilized to generate multiple trajectories of subcellular dynamics on minute-to-hour timescales for a large system of ~10^6^ particles treated implicitly (RDME) and ~10^4^ particles described explicitly (LD) using the one-run-per-GPU approach [62]. These are the experimentally relevant timescales for many biological processes in eukaryotic cells, including mitotic spindle assembly. The 8–12 GB GPU on-board memory is adequate to describe cell dynamic processes involving ~10^6^ particles. For example, when running CellDynaMo on a single contemporary GPU (GeForce GTX 1080), it takes ~70 hours of wall-clock time to generate a single trajectory over 10^6^ steps of the RDME algorithm, which translates to ~30 min of biological time. It takes 3–5 days of computational time to probe the importance of Aurora A enzyme for proper biorientation, or the balance of Aurora B and Phosphatase enzymes on the strength of KT-MT linkages.

### Correct versus incorrect KT-MT attachments

We employed the CellDynaMo framework to interrogate computationally several important aspects of the mitotic spindle assembly process and to revisit and assess the validity of the proposed Search-and-Capture paradigm [8–11,16,18–20]. The results are by no means complete or systematic: this study was mainly devoted to development and testing of the computational modeling framework, and to gaining a few insights about the workings of the Search-and-Capture process with realistic cell geometry and mechanics, as well as kinetics of the most relevant chemical reactions. Surprisingly, even such a limited exploration offers several unexpected insights. We found that, at best, the geometrically and mechanically realistic Search-and-Capture mechanism can result in 2/3 correct (amphitelic) KT-MT attachments (Figs 4–7). The amphitelic attachments usually have ~10 KT-MT linkages (Figs 4–7), which compares well with experimental observations. Cells *in vivo* perform much better [83,84] likely due to the processes we have not included yet into the model (see below); but *in vitro* a similar accuracy was observed [6]. The majority of incorrect attachments are predicted to be merotelic, which agrees with both previous predictions [15] and experimental observations [85]. The reason is that monotelically attached CHs tend to ‘acquire’ more MTs and convert into other KT-MT attachment types. Syntelic attachments, similarly, tend to acquire an MT from the ‘wrong’ pole and become merotelic, or else destabilize and become monotelic again. Merotelic attachments, on the other hand, are hard to correct, and additional attachments do not change their character.

One of the conclusions from this study is intuitively clear: an intermediate rate of KT-MT detachment is optimal for CH connection accuracy. When the MTs detach too rapidly, the amphitelic attachments do not have much chance to stabilize. However, when the MTs do not detach rapidly enough, then incorrect attachments are hard to correct. Our simulations also vividly illustrated the beneficial geometric feedback leading to the characteristic amphitelic attachment scenario: the first attachment rapidly turns the attached KT toward the respective pole, thus shielding this KT partially from the other pole while exposing the other KT more to the other pole. This leads to more attachments biased in the amphitelic way, which improves the spindle angle further. In addition, resulting pulling of the sister KTs apart brings the KT-MT linkers away from the Aurora B cloud, thus reinforcing the correct attachments. On the other hand, one of the greatest ‘dangers’ for a CH that our simulations have revealed is the geometric vicious cycle that occurs when an increasing number of MTs attach to a KT pull this KT toward a pole, thereby exposing it to even larger number of MTs and the other KT to smaller number of MTs. One of the unexpected insights from our study is that CH arms break this vicious cycle by making the CH ‘bulky’ and not allowing it to move rapidly away from the spindle equator, while also allowing both KTs to have a chance to acquire connections from the opposite poles.

Another nontrivial insight from this study is that thermal forces due to the stochastic environment in the cell cytoplasm are important despite bulky CHs: thermal trembling of the MT plus-ends effectively accelerates the KT capture, which poses no danger in the presence of flexible CH arms. It was shown that thermal fluctuations drive random pivoting of microtubules [86], so that the plus-ends of 1-2-μm long microtubules constrained at the minus-end are capable of spanning the 30-degree angle, and, as a result, accelerate the process of kinetochore capturing to just ~3–4 min [86]. In the SRDDM, the 8-μm long microtubules are relatively stiff, and changes in the bending angle do not exceed 2 degrees. However, we found that even this reduced microtubule flexibility is sufficient to enable kinetochore capturing within the first few minutes of computational experiment, in agreement with the effect posited in [86].

We found that the presence of Aurora A can improve the accuracy of formation of KT-MT attachments by breaking the incorrect attachments when a CH is too close to one of the poles, where Aurora A phosphorylates Ndc80 linkers, thus increasing the KT-MT detachment rates and reversing the CH convergence toward the pole. However, at this stage of development, the model is not able to completely reverse the CH movement back to the center of the spindle. This is because the growing MTs impinging on the CH arms bend and slide off the arms rapidly, not exerting any significant polymerization force. This is another valuable insight from our simulations: MT pushing is ineffective and chromokinesins, not yet incorporated into the model, are necessary to generate a pushing force. Lastly, we found that mechanical flexibility of the KTs has little effect on the statistics of KT-MT attachments. However, so far, we tested only relatively stiff KTs that deform only slightly. The logic of hypothesized effects of KT deformation is based on producing large KT deformations, which our simulations cannot currently handle.

A number of important questions remain for the future, including the effects of: 1) excluded volume obstruction of the cellular space by the CH arms, and 2) key molecular motors, such as chromokinesins, kinesin-5, and dynein. In particular, progress in this direction will allow us to explore the ‘anti-poleward wind’ concept and formation of the spindle interpolar bundles [87–89], not to mention the mechanisms proposed as alternatives to Search-and-Capture. A pioneering study [90] paved the way for understanding the roles of molecular motors in spindle maintenance, examining conditions under which antiparallel microtubules overlapping can stabilize and maintain a finite distance between the spindle poles [90]. As the spindle assembly process is complex enough, in this study we fixed the spindle pole positions. In the future, using SRDDM, we will be able to interrogate the role of molecular motors in full three-dimensional spindle evolution. Recent data suggests that KTs do not become captured by the end-on MT attachments right away; rather, long centrosomal MTs interact with CHs laterally by bringing CHs to the spindle surface, where the lateral attachments are converted into the end-on ones [91]. Utilizing the CellDynaMo package will enable us to directly simulate this phenomenon. When the formation of the spindle surface is disrupted, the assembly likely reverts to the Search-and-Capture mechanism, and so a thorough investigation of this mechanism in future reports is still valuable.

## Supporting information

S1 Text Supplemetary Methods. Supplementary Movies. Table A in S1 Text. Physical parameters used in Stochastic Reaction-Diffusion-Dynamics Model. Fig A in S1 Text. Illustration of the numerical algorithms implemented in CellDynaMo for simulations of molecular transport. Fig B in S1 Text. Flexibility of chromosome arms. Fig C in S1 Text. Force field parameterization for cohesion ring. Fig D in S1 Text. Dynamics of MT assembly and disassembly and likelihoods of MT catastrophe and rescue using CellDynaMo. Fig E in S1 Text. Histograms of pushing time intervals, pulling forces, and pushing forces. Fig F in S1 Text. Benchmark tests for Langevin Dynamics algorithm implemented in CellDynaMo. Fig G in S1 Text. Benchmark test for one-dimensional translational diffusion and one-dimensional rotational diffusion in Langevin Dynamics algorithm implemented in CellDynaMo. Fig H in S1 Text. Benchmark test for diffusion component of RDME algorithm implemented in CellDynaMo. Fig I in S1 Text. Benchmark tests for kinetics component of RDME algorithm implemented in CellDynaMo. Fig J in S1 Text. Influence of stochastic noise and centromere flexibility on types of KT-MT attachments and KT-KT distance.
(PDF)

S1 Movie Fast KT-MT dissociation and soft spring connecting KTs exhibits improvement. (MP4)

S2 Movie Effect of chromosome arms on final CH position and orientation. (MP4)

S3 Movie Modeling flexible KT surface. (MP4)
