# Supplementary material for: CellDynaMo–stochastic reaction-diffusion-dynamics model: Application to search-and-capture process of mitotic spindle assembly
Source: PLoS Comput Biol. 2022 Jun 3;18(6):e1010165. doi: 10.1371/journal.pcbi.1010165 (PMC9200364; doi:10.1371/journal.pcbi.1010165)
Supplement: S1 Text — Supplementary Movies. Table A in S1 Text. Physical parameters used in Stochastic Reaction-Diffusion-Dynamics Model. Fig A in S1 Text. Illustration of the numerical algorithms implemented in CellDynaMo for simulations of molecular transport. Fig B in S1 Text. Flexibility of chromosome arms. Fig C in S1 Text. Force field parameterization for cohesion ring. Fig D in S1 Text. Dynamics of MT assembly and disassembly and likelihoods of MT catastrophe and rescue using CellDynaMo. Fig E in S1 Text. Histograms of pushing time intervals, pulling forces, and pushing forces. Fig F in S1 Text. Benchmark tests for Langevin Dynamics algorithm implemented in CellDynaMo. Fig G in S1 Text. Benchmark test for one-dimensional translational diffusion and one-dimensional rotational diffusion in Langevin Dynamics algorithm implemented in CellDynaMo. Fig H in S1 Text. Benchmark test for diffusion component of RDME algorithm implemented in CellDynaMo. Fig I in S1 Text. Benchmark tests for kinetics component of RDME algorithm implemented in CellDynaMo. Fig J in S1 Text. Influence of stochastic noise and centromere flexibility on types of KT-MT attachments and KT-KT distance. (PDF) [file pcbi.1010165.s001.pdf]

CellDynaMo – Stochastic Reaction-Diffusion-Dynamics Model: Application to Search-and-Capture Process of Mitotic Spindle Assembly

Supplementary Information

Evgenii Kliuchnikov[1], Artem Zhmurov[2], Kenneth A. Marx[1], Alex Mogilner[3*], Valeri Barsegov[1*]

[1]Department of Chemistry, University of Massachusetts, Lowell, Massachusetts, United States of America, [2]KTH Royal Institute of Technology, Stockholm, Sweden, [3]Courant Institute for Mathematical Sciences and Department of Biology, New York University, New York, New York, United States of America

Supplementary Methods

Benchmark tests for Langevin Dynamics algorithm: In CellDynaMo, all the mechanical components are represented by single interaction centers (beads) or as systems of beads connected by harmonic springs. To assess the accuracy of numerical implementation of the Langevin Dynamics, we carried 1-μs long simulation runs (20,000 steps of integration) for a small system of three beads connected by the harmonic springs (see Fig F-A). In this setup, all three beads are in the xy-plane; beads 1 and 2 are fixed, and a constant pulling force $\boldsymbol{f}$ is applied to bead 3 (the force vector is collinear to the xy-plane). For this system in two dimensions, the forces and the displacements can be calculated analytically using the Newton's equations in the x- and y-dimensions:

$$\begin{cases} f_x = f_{13}\cos\varphi - f_{23}\cos\psi = 0 \\ f_y = f - f_{13}\sin\varphi - f_{23}\sin\psi = 0 \end{cases} \tag{1}$$

In Eqs 1 above, f_x and f_y are the $x-$ and $y-$ components, respectively, of the total force $\boldsymbol{f}$ applied to bead 3 (tagged), f_{13} and f_{23} are the restoring forces with which the first and second springs pull on bead 3, φ is the angle formed by beads 2, 1, and 3, and ψ is the angle formed by beads 1, 2, and 3 (see Fig F-A). The forces and displacements are related through Hooke's law:

$$f_{13} = \kappa_{13}\Delta x_{13} \text{ and } f_{23} = \kappa_{23}\Delta x_{23} \tag{2}$$

where κ_{13} and κ_{23} are the spring constants of the springs which connect beads 1 and 3, and beads 2 and 3. Δx_{13} and Δx_{23} are distances between beads 1 and 3, and beads 2 and 3, respectively. By substituting the expressions for f_{13} and f_{23} from Eqs 2 into Eqs 1, we obtain the exact expressions for the beads' displacements:

$$\Delta x_{13} = \frac{f}{\kappa_{13}(\cos\varphi\tan\psi + \sin\varphi)} \text{ and } \Delta x_{23} = \frac{f}{\kappa_{23}(\cos\psi\tan\varphi + \sin\psi)} \tag{3}$$

In the benchmark tests for different values of the applied force $f = 5$ pN and 50 pN, we set the initial values for the angles to $\varphi_0 = 26.59°$ and $\psi_0 = 18.45°$, and the spring constants to $\kappa_{13} = \kappa_{23} = 10$ pN/nm. For 5 pN force, the equilibrium (final) values of the angles were $\varphi = 26.76°$ and $\psi = 18.58°$, and for 50 pN force, the equilibrium values came to $\varphi = 28.36°$ and $\psi = 19.72°$. These were used to calculate the

exact values of extensions. The time profiles of displacements Δx_{13} and Δx_{23} are displayed in Fig F, which show excellent agreement between the exact and numerical results.

Dependence of numerical error on integration timestep: To investigate the dependence of the numerical error on the integration timestep $\delta t = 5\times10^{-2}$-$5\times10^{2}$ ps, we calculated and compared for the three-bead system (Fig F) the asymptotic values of the particle displacements obtained for the applied 50-pN pulling force with the CellDynaMo implementation (Δx_{sim}) and using the exact analytical expression (Δx_{exact}) for Δx_{13} and Δx_{23} (Fig F). The relative error, $Err(\Delta x) = \frac{|\Delta x_{sim} - \Delta x_{exact}|}{\Delta x_{exact}}$ was found to be very low (<1.8%) in the 5×10^{-2}-5×10^{2} ps range of the integration timestep δt (Fig F-B).

Dependence of numerical error on solution viscosity: To investigate the dependence of numerical error on the cytoplasmic viscosity $\eta = 1$, 5, and 10 cPs and on the integration timestep $\delta t = 5\times10^{-2}$-$5\times10^{2}$ ps, we calculated and compared, for a large system of $N = 100$ Brownian oscillators (with the spring constant $\kappa = 1$ pN/nm), the numerical solution of the average particle displacements obtained with CellDynaMo ($\langle \Delta x(t) \rangle_{sim}$) and the exact analytical expression ($\langle \Delta x(t) \rangle_{exact}$). The exact expression is given by the formula $\langle \Delta x(t) \rangle = \frac{f}{\kappa}(1 - e^{-\kappa t/\gamma})$, where $\gamma = 6\pi\eta R$ is the friction coefficient, η is the viscosity, and R is the particle size (see Fig F). First, we varied the viscosity but fixed the integration timestep $\delta t = 50$ ps. The numerical and analytical results practically collapse on the same curve (Fig F-C), and the relative error, $Err(\langle \Delta x(t) \rangle) = \frac{|\langle \Delta x(t) \rangle_{sim} - \langle \Delta x(t) \rangle_{exact}|}{\langle \Delta x(t) \rangle_{exact}}$, was found to be very low <0.4% (see *the inset* to Fig F-C) for all viscosity values. Next, we fixed the viscosity at $\eta = 1$ cPs and varied the integration timestep δt over 4 orders of magnitude from 5×10^{-2} to 5×10^{2} ps. The relative error was found to be very low <0.3% (see Fig F-D), and the average relative error was <0.16% (see *the inset* to Fig F-D). Hence, our choice of $\delta t = 50$ ps as the timestep for Langevin Dynamics based description of the mechanical and force-dependent processes is reasonable.

Benchmark test for translational and rotational diffusion in Langevin Dynamics: To examine the system's thermal fluctuations, we compared the numerical implementations of the one-dimensional translational diffusion for a cylinder along the x-axis (see the *inset* in Fig G) and one-dimensional rotational diffusion for a cylinder around the z-axis (see the *inset* in Fig G) for a single KT pair (centromere; CH), approximated by a cylinder, obtained with the CellDynaMo package and with the exact analytical expressions, $D_x = \frac{k_B T \cdot \ln(L_{CH}/d_{CH})}{4\pi\eta L_{CH}}$ and $D_\theta = \frac{k_B T \cdot 3\ln(L_{CH}/d_{CH})}{\pi\eta L_{CH}^3}$, where k_B is the Boltzmann's constant, T = 300 K, η is the solution viscosity set to be equal to the viscosity of water ($\eta = 1$ cPs), and $L_{CH} = 4R_{CH} = 1.450$ µm is the length and $d_{CH} = 2R_{CH} = 0.725$ µm is the diameter of the centromere. We carried out three 2.5-min independent runs for a single centromere. In these test simulations, the MT dynamics was turned off, and the integration timestep δt was decreased from 50 ps to 5 ps to make thermal fluctuations more noticeable. The time profiles of $\langle \Delta x^2 \rangle / 2t$ and $\langle \Delta \theta^2 \rangle / 2t$ are displayed in Fig G The obtained asymptotic values of 0.154 µm^2/s and 0.866 rad^2/s compare well with the exact analytical solutions for $D_x = 0.152$ µm^2/s and $D_\theta = 0.869$ rad^2/s, respectively (see Fig G).

Benchmark test for diffusion component of RDME algorithm: To assess the accuracy of numerical implementation of the diffusion part of RDME, we placed 10^4 molecules of AB in the center of the cell (central subcell), $x_0 = 0$, at the initial time $t = 0$, as displayed in Fig A and observed spreading of AB particles at later time points $t = 1$, 2, 5, 10, and 20 s. The non-parametric density estimates of the distributions of particles displacements in one dimension, constructed using the numerical output from

CellDynaMo simulations, are compared with the theoretical curves of the exact probability distributions of particles' displacements,

$$P(x,t|x_0) = (4\pi Dt)^{-\frac{3}{2}} exp\left[\frac{(x-x_0)^2}{4Dt}\right] \quad (4)$$

in Fig H, which shows excellent agreement between the numerical and exact analytical results. In Eq 4, $D = 7.3\times10^7$ nm^2/s is the diffusion constant (Fig H) for AB molecules, which was estimated using the Einstein-Stokes formula, $D = 6\pi\eta R_A$, where η is the viscosity set to be equal to the viscosity of water ($\eta = 1$ cPs) and $R_A = 2.9$ nm is the size of the AB molecule (see Table A). Therefore, the results obtained indicate excellent agreement between the exact description of the Brownian diffusion (Eq 4) and the numerical description of the Brownian diffusion implemented in the CellDynaMo package.

Benchmark tests for kinetics component of RDME algorithm: *Consecutive two-step irreversible kinetics* is described by the following scheme, $A \overset{k_1}{\to} B \overset{k_2}{\to} C$, with species A, B and C and the reaction rate constants for the first step ($A \longrightarrow B$) k_1 and the second step ($B \longrightarrow C$) k_2. The populations p_A, p_B, and p_C of species A, B and C are described by the following coupled ordinary differential equations:

$$\frac{dp_A}{dt} = -k_1 p_A \quad (5)$$

$$\frac{dp_B}{dt} = k_1 p_A - k_2 p_B \quad (6)$$

$$\frac{dp_C}{dt} = k_2 p_B \quad (7)$$

The exact analytical expressions for the time-dependent populations $p_A(t)$, $p_B(t)$, and $p_C(t)$ are the following:

$$p_A(t) = e^{-k_1 t} \quad (8)$$

$$p_B(t) = \frac{k_1}{k_2 - k_1}(e^{-k_1 t} - e^{-k_2 t}) \quad (9)$$

$$p_C(t) = 1 - e^{-k_1 t} - \frac{k_1}{k_2 - k_1}(e^{-k_1 t} - e^{-k_2 t}) \quad (10)$$

Single-step reversible kinetics is described by the scheme $A \rightleftharpoons B$, with the reaction rate constants for the forward step ($A \longrightarrow B$), k_1, and the backward step ($B \longrightarrow A$), k_{-1}. The populations p_A and p_B, of states A and B are described by the following coupled ordinary differential equations:

$$\frac{dp_A}{dt} = -k_1 p_A + k_{-1} p_B \quad (11)$$

$$\frac{dp_B}{dt} = k_1 p_A - k_{-1} p_B \quad (12)$$

The exact analytical expressions for the time-dependent populations $p_A(t)$ and $p_B(t)$ are:

$$p_A(t) = \frac{k_{-1} + k_1 e^{-(k_1 + k_{-1})t}}{k_1 + k_{-1}} \quad (13)$$

$$p_B(t) = \frac{k_1 - k_1 e^{-(k_1 + k_{-1})t}}{k_1 + k_{-1}} \quad (14)$$

Eqs 8, 9, and 10 for the consecutive two-step irreversible kinetics and Eqs 13 and 14 for the single-step reversible kinetics were used in benchmark simulations to compare the exact description of chemical kinetics with the numerical description of kinetics implemented in the CellDynaMo package.

Flexibility of chromosomes: To model flexible chromosomes, in the CellDynaMo package the chromosome arms are represented as a collection of spherical beads (see Fig 6B in the main text and Fig C) of 362.5-nm radius. Each pair of beads is connected by a harmonic spring characterized by the stretching rigidity $K_{CH,r}$ (Table A), and each triplet of connected beads is described by the bending rigidity $K_{CH,\theta}$ (Table A). We parameterized the chromosome flexibility by setting the stretching rigidity to $K_{CH,r} =$ 3.3×10^3 pN/nm (Table A) and by varying the bending rigidity ($K_{CH,\theta}$) in the bending potential U_{CH}^{bend} (see Eq 5 in the main text). In the test LD simulations, we set $K_{CH,\theta}$ to be equal to 2.5×10^2 kJ/mol·rad^2 (flexible chromosome arms), to 2.5×10^5 kJ/mol·rad^2 (semi-flexible chromosome arms), and to 2.5×10^8 kJ/mol·rad^2 (stiff chromosome arms), and we ran ~1,000 equilibrium LD trajectories for each value of $K_{CH,\theta}$. Using the simulation output, we constructed the nonparametric density estimates (see Methods in the main text) of the distributions of bending angle $\Delta\theta$, $P(\Delta\theta)$, for $K_{CH,\theta} =$ 2.5×10^2, 2.5×10^5, and 2.5×10^8 kJ/mol·rad^2, which are compared in Fig B. Based on published experimental studies, which report the 1-5 degree range of bending-angle fluctuations [1], we selected $K_{CH,\theta} =$ 2.5×10^5 kJ/mol·rad^2 to characterize the flexural rigidity of the chromatids within the same chromosomes.

Bending constraints imposed by cohesin rings: To model cohesin rings constraining the movement of chromosome arms in the CellDynaMo package, we represented the cohesin rings by weak harmonic springs that link flexible chromatid arms together (Fig 1B in the main text and Fig C-C). Because the chromatid arms are described using the bead-spring representation, these weak harmonic springs connect the corresponding beads in different chromatids (Fig 1B in the main text, Fig C-C). We considered chromosomes with two different values of the contour lengths: 4 μm and 8 μm. We parametrized the fluctuations in the chromosome width by varying the stretching rigidity for cohesin rings (K_{coh}) in the stretching potential U_{coh}^{str} (Eq 5 in the main text). In the test LD simulations, we set $K_{coh,r}$ to be equal to 10^{-3} kJ/(mol·nm^2) (soft cohesin rings) and to 10^3 kJ/(mol·nm^2) (stiff cohesin rings), and we ran ~1,000 equilibrium LD trajectories for each value of $K_{coh,r}$. Using the simulation output, we constructed the nonparametric density estimates (see Methods in the main text) of the distribution of the separation distances Z, $P(Z)$, for $K_{coh,r} =$ 10^3 kJ/(mol·nm^2) and 10^{-3} kJ/(mol·nm^2), which are compared in Fig C-A,B. Experimental studies show that chromatid ends separate from each other by distances not exceeding 2 μm [2]. Therefore, we selected $K_{coh,r}$ =10^{-3} kJ/(mol·nm^2) to model fluctuations in the chromosome width.

Supplementary References:

1. Houchmandzadeh B, Marko JF, Chatenay D, Libchaber A. Elasticity and structure of eukaryote chromosomes studied by micromanipulation and micropipette aspiration. J Cell Biol. 1997;139: 1–12.

2. Nasmyth K, Haering CH. The structure and function of SMC and kleisin complexes. Annu Rev Biochem. 2005;74: 595–648.

3. Silkworth WT, Nardi IK, Paul R, Mogilner A, Cimini D. Timing of centrosome separation is important for accurate chromosome segregation. Mol Biol Cell. 2012;23: 401–411.

4. Alieva IB, Uzbekov RE. Where are the limits of the centrosome? Bioarchitecture. 2016;6: 47–52.

5. Decker M, Jaensch S, Pozniakovsky A, Zinke A, O'Connell KF, Zachariae W, et al. Limiting amounts of centrosome material set centrosome size in C. elegans embryos. Curr Biol. 2011;21: 1259–1267.

6. Wollman R, Cytrynbaum EN, Jones JT, Meyer T, Scholey JM, Mogilner A. Efficient chromosome capture requires a bias in the "search-and-capture" process during mitotic-spindle assembly. Curr Biol. 2005;15: 828–832.

7. McIntosh JR, Landis SC. The distribution of spindle microtubules during mitosis in cultured human cells. J Cell Biol. 1971;49: 468–497.

8. Kononova O, Kholodov Y, Theisen KE, Marx KA, Dima RI, Ataullakhanov FI, et al. Tubulin bond energies and microtubule biomechanics determined from nanoindentation in silico. J Am Chem Soc. 2014;136: 17036–17045.

9. Ris H, Witt PL. Structure of the mammalian kinetochore. Chromosoma. 1981;82: 153–170.

10. Drpic D, Almeida AC, Aguiar P, Renda F, Damas J, Lewin HA, et al. Chromosome Segregation Is Biased by Kinetochore Size. Curr Biol. 2018;28: 1344–1356.

11. Wei RR, Sorger PK, Harrison SC. Molecular organization of the Ndc80 complex, an essential kinetochore component. Proc Natl Acad Sci. 2005;102: 5363–5367.

12. Ciferri C, Pasqualato S, Screpanti E, Varetti G, Santaguida S, Dos Reis G, et al. Implications for Kinetochore-Microtubule Attachment from the Structure of an Engineered Ndc80 Complex. Cell. 2008;133: 427–439.

13. Wang H-W, Long S, Ciferri C, Westermann S, Drubin D, Barnes G, et al. Architecture and flexibility of the yeast Ndc80 kinetochore complex. J Mol Biol. 2008;383: 894–903.

14. Elkins JM, Santaguida S, Musacchio A, Knapp S. Crystal structure of human Aurora B in complex with INCENP and VX-680. J Med Chem. 2012;55: 7841–7848.

15. Walker RA, O'Brien ET, Pryer NK, Soboeiro MF, Voter WA, Erickson HP, et al. Dynamic instability of individual microtubules analyzed by video light microscopy: rate constants and transition frequencies. J Cell Biol. 1988;107: 1437–1448.

16. Scott DW. Scott's rule. Wiley Interdiscip Rev Comput Stat. 2010;2: 497–502.

Supplementary Movies

S1 Movie. Fast KT-MT dissociation and soft spring connecting KTs exhibits improvement: This movie shows 15 minutes of cell life and is related to Figs 4 and 7A,7C and 7E in the main text. The cell includes a single KT pair (blue) and centrosomes each having 1,500 MTs (lime). The KT surface area is 0.5 μm^2 (orange). The pushing/pulling force per MT is 10 pN/10 pN, and the KT-MT dissociation rate is $(0.1–2.9)\times10^{-1}$ s^{-1}. A KT pair is placed in the equatorial plate of the cell with the KT-pair axis coinciding with the z-axis (perpendicular to the direction of biorientation to eliminate KT attachment bias to either CS). This configuration is optimal for formation of merotelic attachments; yet, due to rapid dissociation of the KT-MT complex and soft spring connecting KTs, the majority of trajectories show the amphitelic attachments. The movie consists of two parts. The first part of the movie shows a side-view of the cell for better observation of KT movements, whereas the second part of the move shows the KT-MT interface in more detail. The length of the movie is 1 min (the movie is played 30 times faster than the process).

S2 Movie. Effect of chromosome arms on final CH position and orientation: This movie shows 15 minutes of cell life and is related to Figs 6A-D and 7B, 7D and 7F in the main text. The cell includes one CH with arms (blue); each centrosome has 1,500 MTs (lime). The KT surface area is 0.5 μm^2 (orange). The pushing/pulling force per MT is 10 pN/10 pN, and the KT-MT dissociation rate is $(0.1–2.9)\times10^{-1}$ s^{-1}. A CH is placed in the equatorial plate of the cell with the CH axis coinciding with the z-axis (perpendicular to the direction of biorientation to eliminate KT attachment bias to either CS). The inclusion of CH arms did not influence the final CH position and orientation. The movie consists of two parts. The first part of the movie shows a view from the top of the cell for better observation of the CH movement along the spindle-axis. The second part of the movie sheds light on the overall 3D picture. The length of the movie is 1 min (the movie is played 30 times faster than the process).

S3 Movie. Modeling flexible KT surface: The movie shows 15 minutes of cell life and is related to Fig 6E-G and in the main text and Fig J-D. The cell includes a single KT pair (blue) and centrosomes each having 1,500 MTs (lime). The KT surface area is 0.5 μm^2 (orange). The pushing/pulling force per MT is 10 pN/10 pN, and the KT-MT dissociation rate is $(0.1–2.9)\times10^{-1}$ s^{-1}. A KT pair is placed in the center of the cell with the KT-pair axis pointing is the x-direction (direction of biorientation to eliminate KT attachment bias to either CS). The movie consists of two parts. The first part of the movie shows a side-view of the cell for better observation of KT movements. The second part of the move shows the zoomed-in KT-MT interface for a more detailed deformation observation. The length of the movie is 1 min (the movie is played 30 times faster than the process).

Table A. Physical parameters used in Stochastic Reaction-Diffusion-Dynamics Model: Listed are numerical values of model parameters, which define: cell morphology (shape/curvature, size/length, surface area, copy number of molecules, distance between components, etc.), biochemical kinetics and molecular transport (size of subcells and time step used in RDME approach), MT dynamics (growth/shortening rate, frequency of catastrophe/rescue) and dynamic cell evolution (time step, temperature, viscosity, bending rigidities, stiffness, pulling and pushing force) used in LD approach.

Parameters	Value, units	Description	Reference
**Parameters of Cell Morphology**			
d_{CS}	10 µm	distance between CSs	[3]
R_{CS}	400 nm	CS bead size	[4,5]
N_{MT}	750	number of MTs per CS	[6,7]
R_{MT}	12 nm	MT bead size	[8]
N_{KT}	2, 4, 6, 8, 10	number of KTs (2 KTs per single CH)	
d_{KT}	725 nm	KT-KT equilibrium distance	[9]
A_{KT}	0.15 µm^2	KT surface area	[10]
χ	0 – 1	KT surface curvature	
R_{CH}	362.5 nm	size of beads representing KT and Ch	[9]
L	varied	Ch contour length	
N_{Ndc}	750	number of Ndc80 per KT surface	
l_{Ndc}	65 nm	size of Ndc80-complex	[11,12]
R_{Ndc}	4 nm	size of Ndc80 kinetochore-associated domain	[13]
a	8 µm	for ellipsoidal shape, a is semi-major axis, b and c are semi-minor axes; for rectangular shape, a, b, and c are length, width, and height	
b	5 µm		
c	5 µm		
**RDME parameters**			
l_{SV}	250 nm	size of subcells	
τ	4.3×10^{-5} s	time step	
R_A	2.9 nm	size of Aurora B	[14]
P:A ratio	0.1	Phosphatase to Aurora B ratio	
**MT dynamics parameters**			
v_{gr}	7.5 µm/min	MT growth rate	[15]
v_{sh}	27 µm/min	MT shortening rate	[15]
ω_{cat}	2.5×10^{-3} s^{-1}	catastrophe frequency	[15]
ω_{res}	3.0×10^{-2} s^{-1}	rescue frequency	[15]
**Langevin Dynamic parameters**			
δt	50 ps	time step	
T	300 K	Temperature	
η	1 cPs	Viscosity	
l_p	4 mm	MT persistence length	[8]
$K_{MT,\theta}$	7.7×10^5 kJ/mol·rad^2	MT bending rigidity	
$\theta_{MT,0}$	180°	equilibrium bending angle for MT	
$K_{MT,r}$	16.7 pN/nm	MT stretching rigidity	
$K_{KT,r}$	3.3×10^3 pN/nm	stretching rigidity for sister KTs	
$K_{CH,r}$	3.3×10^3 pN/nm	Ch stretching rigidity	
$K_{CH,\theta}$	2.5×10^5 kJ/mol·rad^2	Ch bending rigidity	
$\theta_{CH,0}$	180°	equilibrium bending angle for Ch	
$K_{Ndc,r}$	3.1×10^2 pN/nm	Ndc80 stiffness	
$K_{coh,r}$	1.7×10^{-3} pN/nm	Ch-Ch stretching rigidity	
K_{mem}	3.3×10^3 pN/nm	membrane stiffness	
ε	2.1×10^5 kJ/mol	strength of repulsive potential	
f_{push}	10 pN	pushing force per MT	
f_{pull}	10 pN	pulling force per MT	

Supplementary Figures

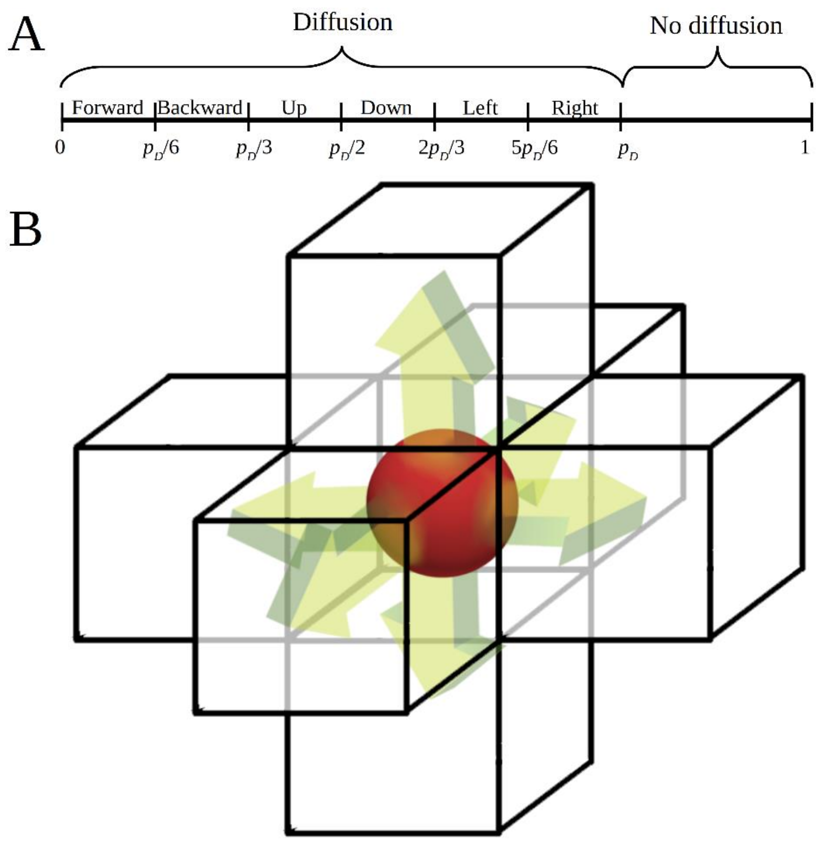

Fig A. Illustration of the numerical algorithms implemented in CellDynaMo for simulations of molecular transport: A) Brownian diffusion of biochemical species (AB and AA enzymes) is modeled by the exchange of particles between the next-neighbor subcell. For each particle, the probability of diffusion p_D is compared with a (pseudo)random number r generated at each time step. If $r < p_D$ the diffusion move is accepted. **B)** Each subcell is surrounded by 6 next-neighbor subcells. The value of p_D determines the direction (forward, backward, up, down, left, and right) and the next-neighbor subcell for particle transfer.

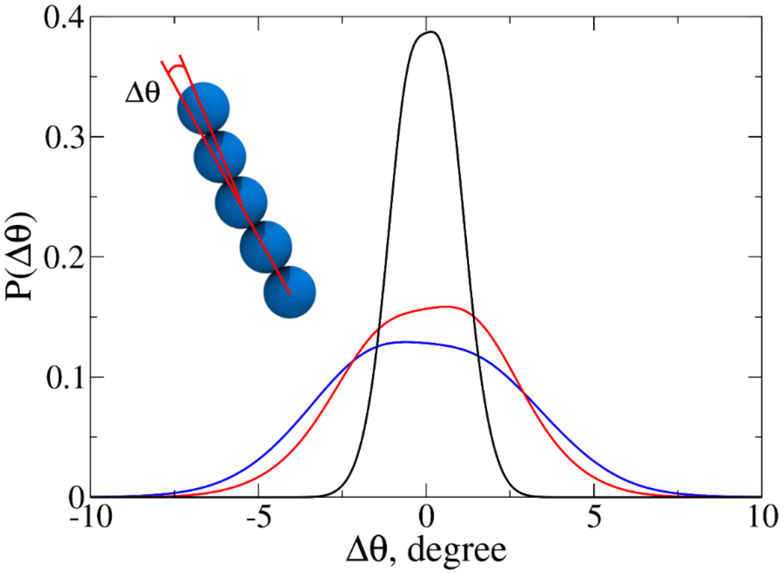

Fig B. Flexibility of chromosome arms: Nonparametric density estimates of the distribution of bending angles $\Delta\theta$, $P(\Delta\theta)$, which characterize the flexural rigidity of a single chromatid, compared for a stiff chromatid ($K_{CH,\theta}$ = 2.5×10^8 kJ/mol·rad^2; black curve), semi-flexible chromatid ($K_{CH,\theta}$ = 2.5×10^5 kJ/mol·rad^2; red curve) and flexible chromatid ($K_{CH,\theta}$ = 2.5×10^2 kJ/mol·rad^2; blue curve). The nonparametric density curves were constructed as described in Ref. [16].

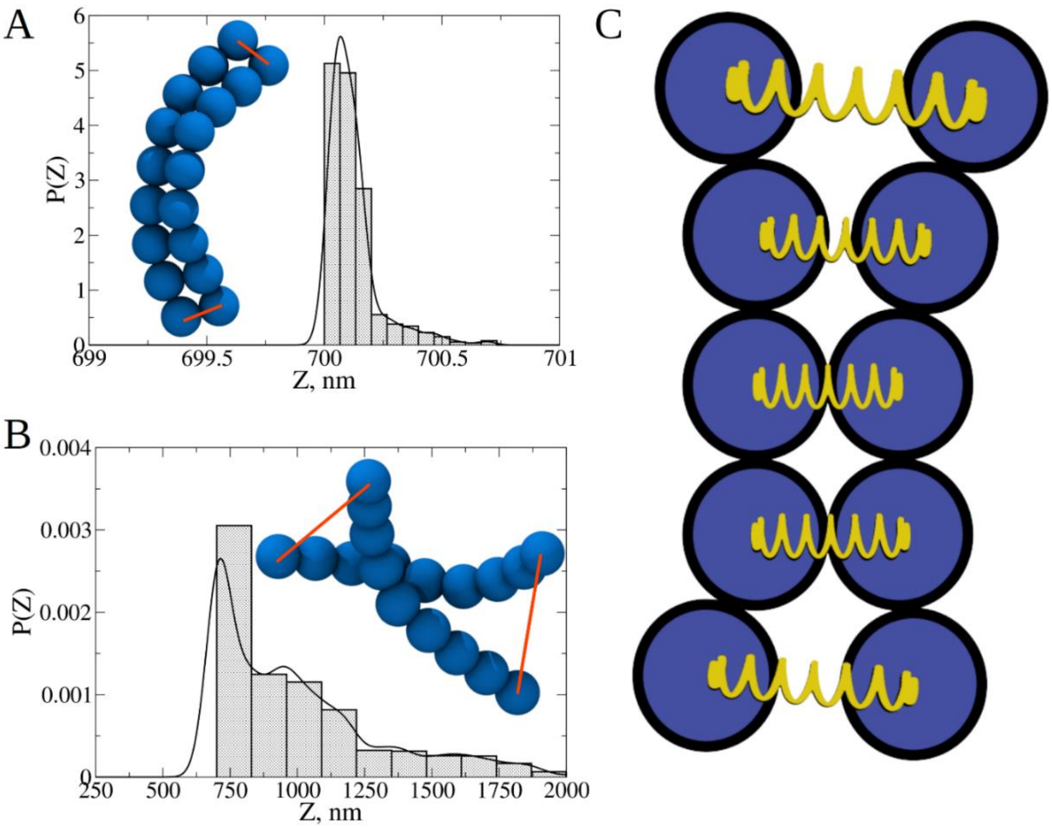

Fig C. Force field parameterization for cohesion ring: A) and **B)** Histogram and nonparametric density-based estimates of the distribution of inter-chromatid distances $P(\mathrm{Z})$ separating the ends of chromatids by distance Z (red lines) within the chromosome (chromosome width) through cohesin rings. The histograms and density curves are compared for $K_{coh,r} = 10^3$ kJ/(mol·nm^2) (stiff cohesin rings; **A**) and for $K_{coh,r} = 10^{-3}$ kJ/(mol·nm^2) (flexible cohesin rings; **B**). The histograms and nonparametric density curves were constructed as described in Ref. [16]. **C**) Idealized schematic of the bending constraints imposed by the presence of cohesion rings connecting the chromosome arms.

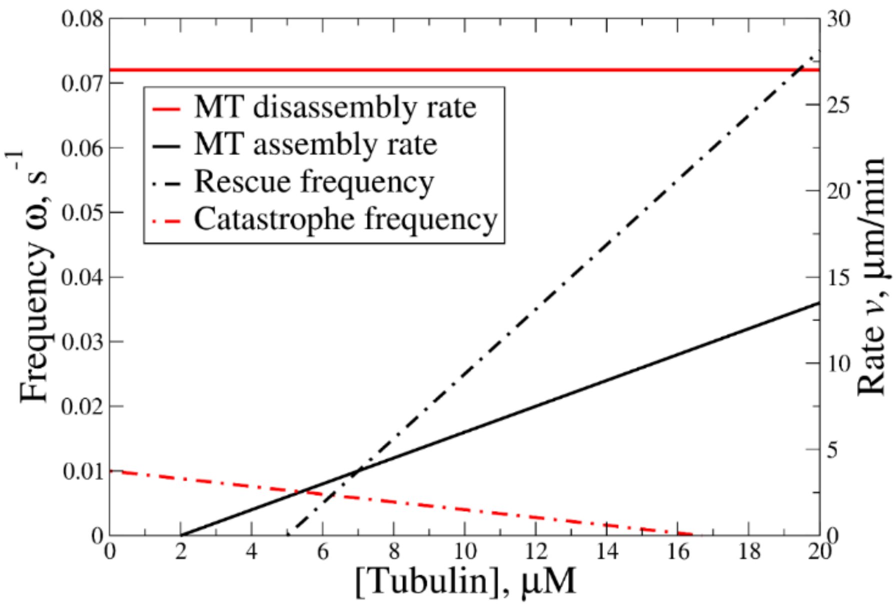

Fig D. Dynamics of MT assembly and disassembly and likelihoods of MT catastrophe and rescue using CellDynaMo: Shown are the experimental rates of MT growth and shortening [15] (solid lines; right y-axis), and experimental frequencies of MT catastrophe and rescue [15] (dash-dotted lines; left y-axis) profiled as functions of molar concentration of the αβ-tubulin dimers. Color denotation is presented in the graph.

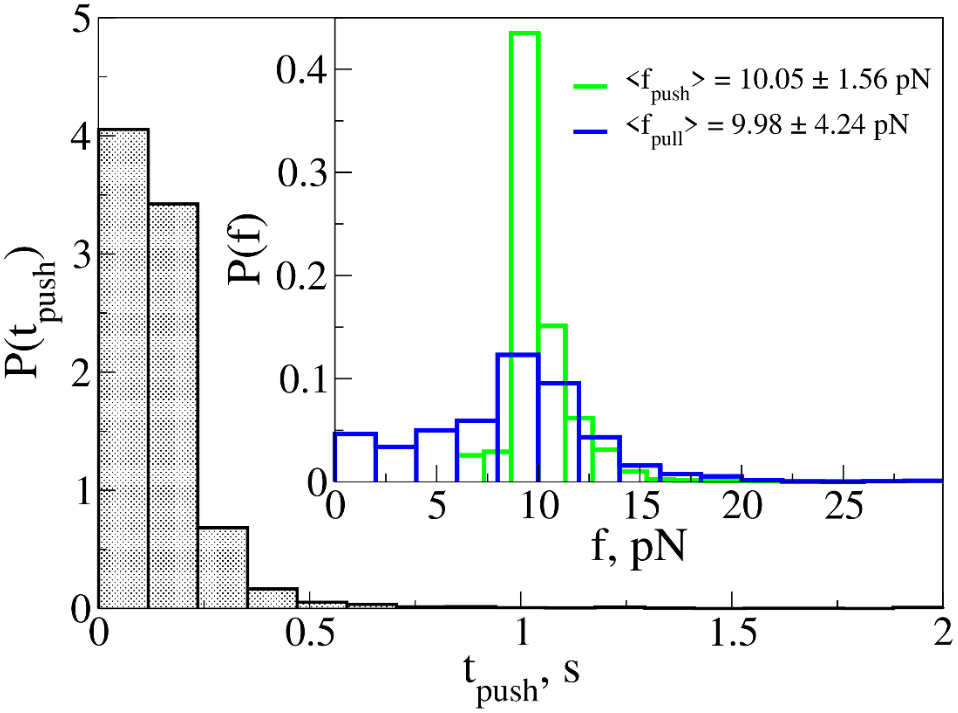

Fig E. Histograms of pushing time intervals, pulling forces, and pushing forces: The histogram-based estimate of the distribution of pushing time intervals $P(t)$ for growing microtubules (grey bars; 10^5 data points). *The inset* shows the histogram-based estimates of the distributions of pushing/pulling forces $P(f)$ (green/blue bars; 10^5 data points) from the growing/shortening MTs obtained from the CellDynaMo based simulations are shown in the *inset*.

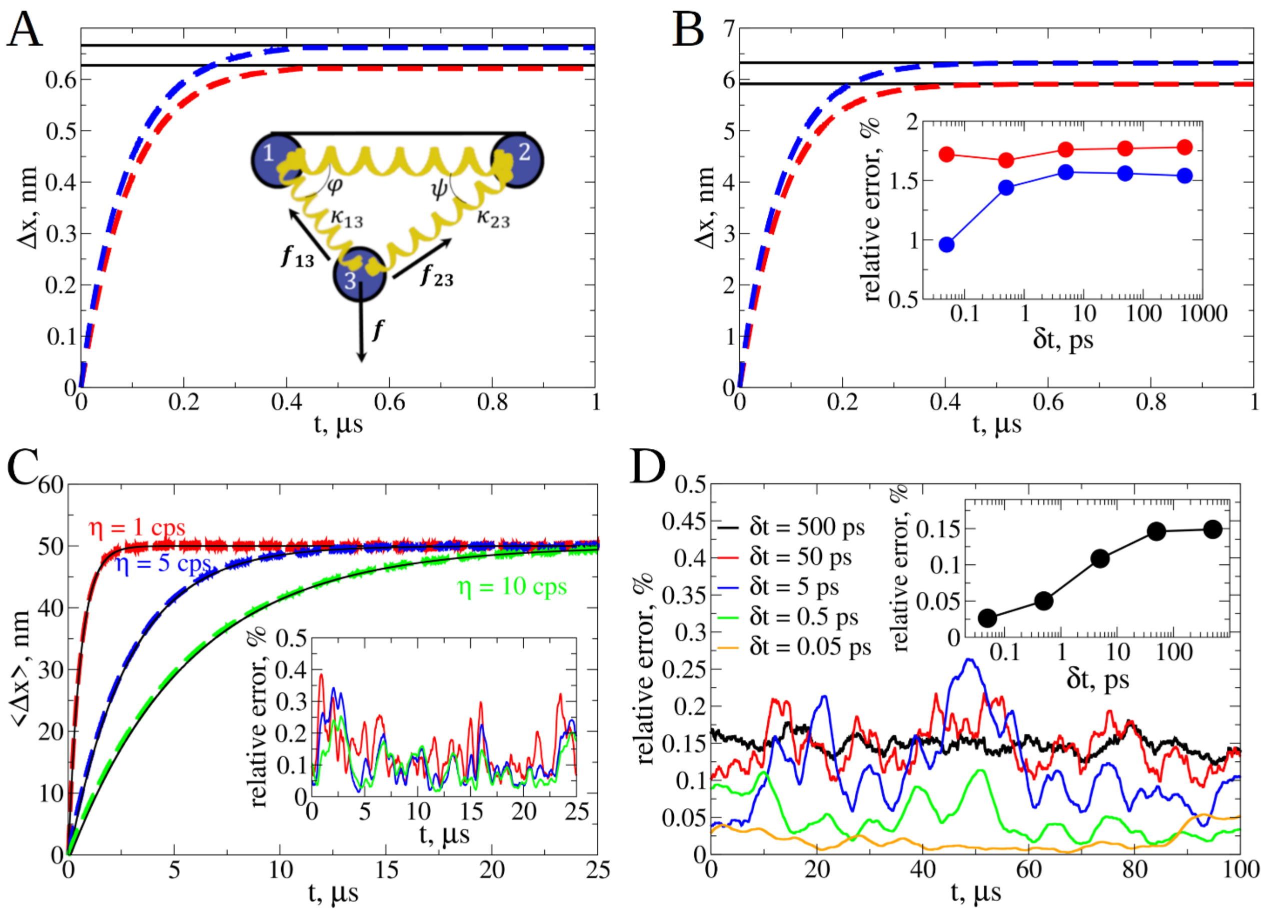

Fig F. Benchmark tests Langevin Dynamics algorithm implemented in CellDynaMo: A) The time-dependent profiles of the spring extensions Δx_{13} (red) and Δx_{23} (blue) obtained numerically using CellDynaMo (dashed lines) and analytically using Eq 3 (solid black lines) for $f = 5$ pN. *The inset* shows the three-bead system with the constrained beads 1 and 2 and tagged bead 3 in the xy-plane (blue balls), which are connected by the harmonic springs (shown in yellow) with the spring constants κ_{13} and κ_{23}, respectively. The tagged bead 3 is pulled with force $\boldsymbol{f}$. The pulling force and the components $\boldsymbol{f_{23}}$ and $\boldsymbol{f_{23}}$ of the restoring force are indicated by black arrows. Also shown are angle φ formed by beads 2, 1, and 3, and angle ψ formed by beads 1, 2, and 3. **B)** The time-dependent profiles of the extensions Δx_{13} (red) and Δx_{23} (blue) obtained numerically using CellDynaMo (dashed lines) and analytically (solid black lines) using Eq 3 for $f = 50$ pN. Relative error of calculation of asymptotic values of Δx_{13} (red lines and data points) and Δx_{23} (blue lines and data points), $Err(\Delta x) = \frac{|\Delta x_{sim} - \Delta x_{exact}|}{\Delta x_{exact}}$ (Δx_{sim} is the asymptotic values of particle displacement from simulations and Δx_{exact} is the value obtained using the exact analytical expression), obtained for different values of the timestep δt are shown in the *inset*. **C)** Time-dependent profiles of the average displacement $\langle \Delta x(t) \rangle$ for the solution viscosity $\eta = 1$ cPs (red color), 5 cPs (blue) and 10 cPs (green) obtained numerically with the integration timestep $\delta t = 50$ ps (dashed curves) and the exact analytical solution (solid curves) for the applied pulling force $f = 50$ pN. *The inset* shows the relative error vs. time profiles. **D)** Time-dependent profiles of the relative error obtained for different integration timesteps: $\delta t = 0.05$ ps, 0.5 ps, 5 ps, 50 ps and 500 ps. The average relative error for different values for the integration timestep δt used are shown in the *inset*.

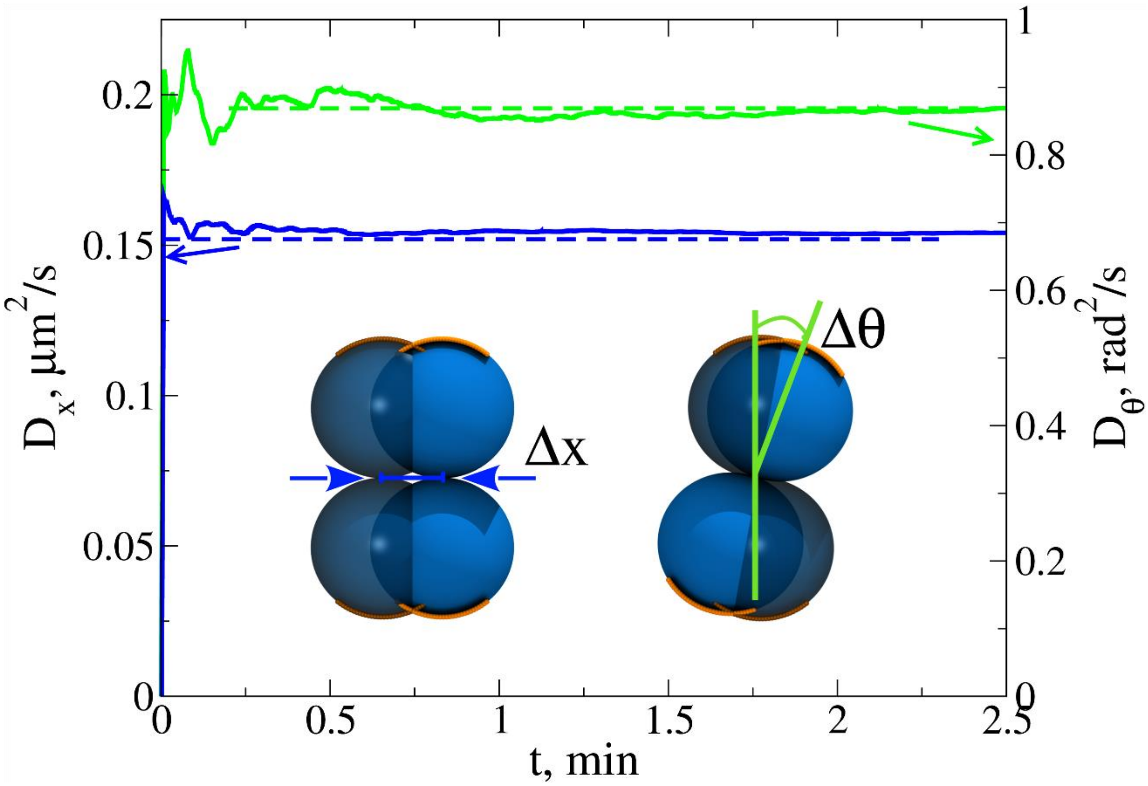

Fig G. Benchmark test simulations for translational and rotational diffusion for Langevin Dynamics in CellDynaMo: Time-dependent profiles of the one-dimensional translational diffusion coefficient $\langle \Delta x^2 \rangle / 2t$ (blue curve) and one-dimensional rotational diffusion coefficient $\langle \Delta \theta^2 \rangle / 2t$ (green curve) for a single KT pair obtained numerically using CellDynaMo. These profiles are compared with the asymptotic values (dashed lines) of these quantities obtained analytically using the formulas $D_x = \frac{k_B T \cdot \ln(L_{CH}/d_{CH})}{4\pi\eta L_{CH}} = 0.152$ µm^2/s and $D_\theta = \frac{k_B T \cdot 3\ln(L_{CH}/d_{CH})}{\pi\eta {L_{CH}}^3} = 0.869$ rad^2/s, respectively. Schematic definitions of the displacement along the x-axis (Δx) and angle of rotation ($\Delta\theta$) in the xy-plane are shown in the *inset*.

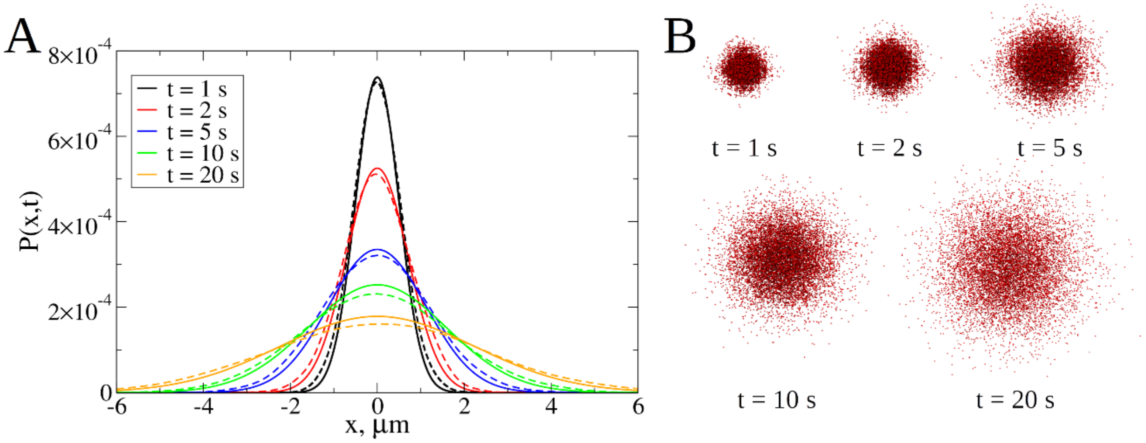

Fig H. Benchmark test for diffusion component of RDME algorithm implemented in CellDynaMo: A) Nonparametric density estimates (dashed lines), obtained using CellDynaMo and the exact theoretical profiles (thin curves), obtained with Eq 4, of the probability density function of the displacements of Brownian particles in one dimension, $P(x, t|x_0)$. The particle is positioned at the origin $x_0 = 0$ at time $t = 0$ are the particles' spreading is compared for the time points $t =$ 1, 2, 5, 10, and 20 s. The nonparametric density curves were constructed as described in Ref. [16]. Color denotation is presented on the graph. **B)** Snapshots of the spatial distributions of a total of 10^4 Brownian particles in three dimensions obtained for different time points, $t =$ 1, 2, 5, 10, and 20 s, which show spreading of the Brownian particles (AB molecules) due to free diffusion.

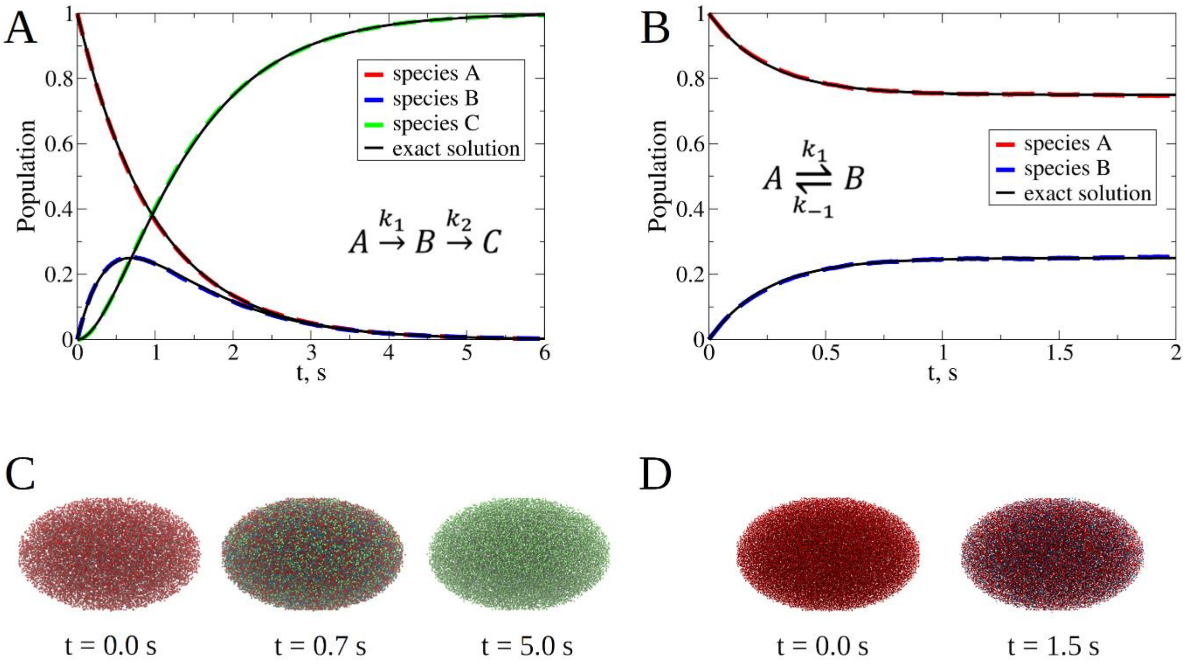

Fig I. Benchmark tests for kinetics component of RDME algorithm implemented in CellDynaMo: **A)** Time-dependent profiles of the populations p_A, p_B, and p_C of chemical species A, B, and C obtained numerically with CellDynaMo and analytically using Eqs 5, 6, and 7 for the two-step consecutive irreversible kinetics. The initial conditions $p_A(0) = 1$, $p_B(0) = 0$, and $p_C(0) = 0$, and the reaction rate constants $k_1 = 1$ s^{-1} and $k_2 = 2$ s^{-1} were used. **B)** Time-dependent profiles of the populations p_A and p_B of chemical species A and B obtained numerically with CellDynaMo and analytically using Eqs 11 and 12 for the single-step reversible kinetics. We used the initial conditions, $p_A(0) = 1$ and $p_B(0) = 0$, and the reaction rate constants $k_1 = 1$ s^{-1} and $k_{-1} = 3$ s^{-1}. Color denotation is presented on the graphs. **C)** Snapshots of the reaction volume for the two-step irreversible kinetics taken at different time points $t =$ 0, 0.7, and 5.0 s, which show the reaction progress (transformation of A into B and formation of C – the end product). **D)** Snapshots of the reaction volume for the reversible kinetics taken at different time points $t = 0$ and 1.5 s, which shows the presence of both species A and B in the equilibrium mixture.

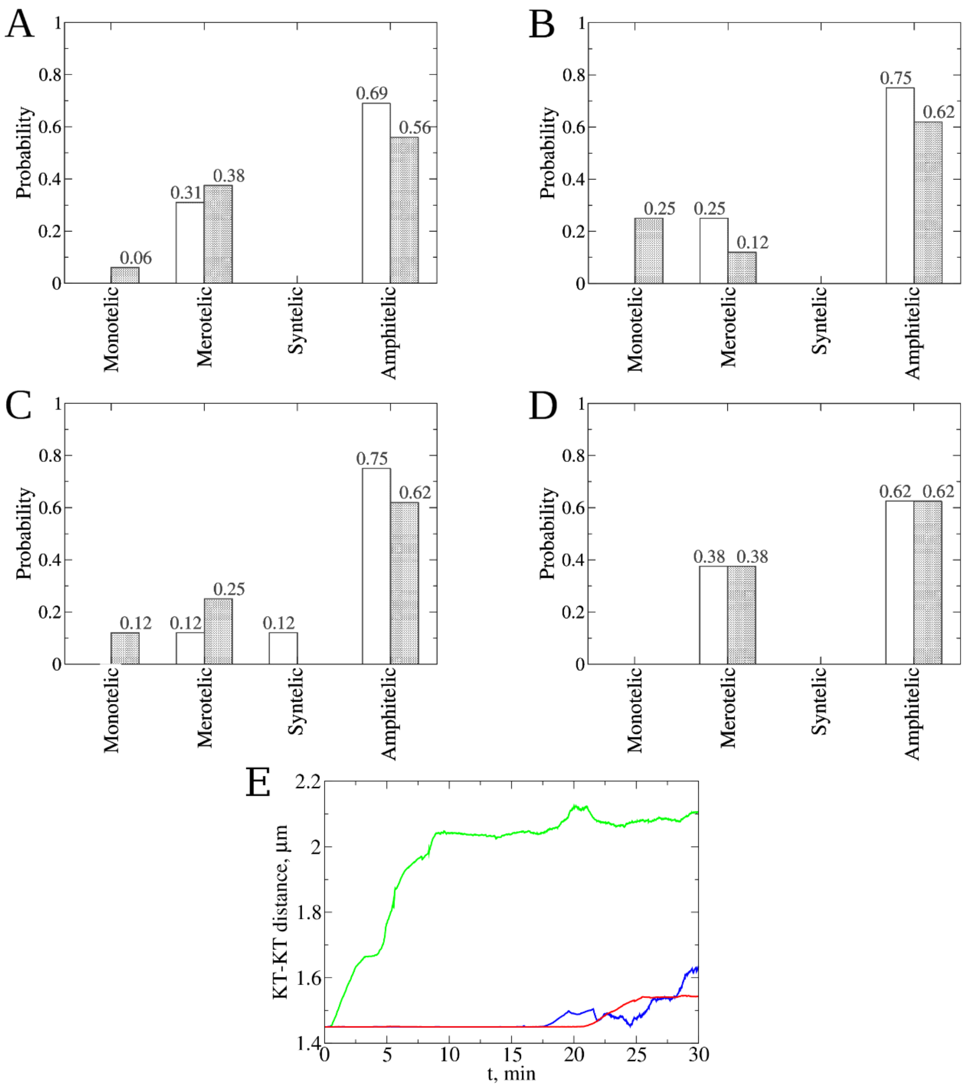

Fig J. Influence of stochastic noise and centromere flexibility on types of KT-MT attachments and KT-KT distance: Comparison of the dynamics of KT-MT attachments (types and number of attachments) from the Stochastic Reaction-Diffusion-Dynamics model based simulations with the random force (shaded bars) and without random force component (blank bars) for: **A**) A single KT pair with the Phosphatase to Aurora B (P:AB) ratio = 1:100, **B**) A single KT pair with the P:AB ratio = 1:10, **C)** A single KT pair with Aurora A present, and **D**) A single KT pair with flexible KT corona surface area (see also Fig 7 in the main text). **E**) Distance vs. time profiles for the case of CHs with chromosome arms. Green, blue and red curves show the typical profiles for the amphitelic, syntelic, and merotelic attachments, respectively.
